# A chalcone derivative reactivates latent HIV-1 transcription through activating P-TEFb and promoting Tat-SEC interaction on viral promoter

**DOI:** 10.1038/s41598-017-10728-w

**Published:** 2017-09-06

**Authors:** Jun Wu, Ming-tao Ao, Rui Shao, Hui-ru Wang, Diao Yu, Mei-juan Fang, Xiang Gao, Zhen Wu, Qiang Zhou, Yu-hua Xue

**Affiliations:** 1 School of Pharmaceutical Sciences and Fujian Provincial Key Laboratory of Innovative Drug Target Research, Xiamen University, Xiamen Fujian, 361005 China; 2 Department of Molecular and Cell Biology, University of California, Berkeley Berkeley, CA94720 USA

## Abstract

The principal barrier to the eradication of HIV/AIDS is the existence of latent viral reservoirs. One strategy to overcome this barrier is to use latency-reversing agents (LRAs) to reactivate the latent proviruses, which can then be eliminated by effective anti-retroviral therapy. Although a number of LRAs have been found to reactivate latent HIV, they have not been used clinically due to high toxicity and poor efficacy. In this study, we report the identification of a chalcone analogue called Amt-87 that can significantly reactivate the transcription of latent HIV provirses and act synergistically with known LRAs such as prostratin and JQ1 to reverse latency. Amt-87 works by activating the human transcriptional elongation factor P-TEFb, a CDK9-cyclin T1 heterodimer that is part of the super elongation complex (SEC) used by the viral encoded Tat protein to activate HIV transcription. Amt-87 does so by promoting the phosphorylation of CDK9 at the T-loop, liberating P-TEFb from the inactive 7SK snRNP, and inducing the formation of the Tat-SEC complex at the viral promoter. Together, our data reveal chalcones as a promising category of compounds that should be further explored to identify effective LRAs for targeted reversal of HIV latency.

## Introduction

The latent HIV reservoir, which contains integrated but transcriptionally silent proviruses, is the principal obstacle to the eradication of HIV/AIDS^1, 2^. Effective curative strategies aiming at eliminating the reservoir are being developed^3–9^. One such strategy, nicknamed “shock and kill”, proposes to reactivate the latent HIV proviruses with latency-reversing agents (LRAs) in the initial “shock” phase. This is followed in the “kill” phase by the use of highly active anti-retroviral therapy (HAART) to prevent new infections and at the same time by making the reactivated cells sensitive to the host immune system and viral cytopathogenicity^10–12^. The implementation of the “shock and kill” strategy has so far been hindered by the lack of clinically effective LRAs. All the available LRAs such as the HDAC inhibitor SAHA, PKC agonist prostratin and BET bromodomain inhibitor JQ1 are either highly toxic or display poor clinical outcomes^13–15^. Thus, high efficacious and specific drugs for reactivating latent HIV are urgently needed.

The reactivation of latent proviral transcription requires the HIV-encoded trans-activator protein Tat, which stimulates the elongation stage of RNA polymerase (Pol) II transcription to produce the full-length viral transcripts^14^. Tat acts by recruiting the host super elongation complex (SEC) containing CDK9, cyclin (Cyc)T1, AFF1 or AFF4, ELL1 or ELL2, and AF9 or ENL to the viral TAR RNA element, which is a stem-loop structure formed at the 5′ end of the nascent HIV transcript^16, 17^. Once recruited, the SEC components CDK9 and CycT1, which forms a heterodimer referred to as P-TEFb, phosphorylates the C-terminal domain of the largest subunit of Pol II and a pair of negative elongation factors DSIF and NELF, leading to the release of Pol II from promoter-proximal pausing. On the other hand, the ELL1/2 subunit of the SEC can also directly stimulate the processivity of Pol II by suppressing transient pausing. Thus, working from a single Tat-SEC complex, P-TEFb and ELL1/2 can synergistically activate Pol II elongation along the provial DNA to reverse HIV latency^18–21^.

In latently infected T cells, the vast majority of P-TEFb is sequestered in a catalytically inactive complex called the 7SK snRNP^4^. In addition to P-TEFb, this 7SK snRNP also contains the 7SK snRNA that acts as a molecular scaffold to hold the complex together and the HEXIM protein, which functions as an inhibitor of CDK9^17, 22–25^. Within the 7SK snRNP, the-La related protein LARP7 and the 7SK snRNA capping enzyme MePCE are also constitutive components, which stabilize the 7SK snRNA and may also be involved in regulating the release of P-TEFb^26–29^. The sequestration of P-TEFb in 7SK snRNP keeps the overall P-TEFb/SEC activity in a cell at a very low level and has been proposed as a key factor contributing to HIV latency^26^.

Chalcones, also known as chalconoids, are aromatic ketones that contain two phenyl rings and often emerge as intermediates in the synthesis of many biological compounds. They have been shown to possess an array of biological activities including anti-inflammatory, antioxidant, antitumor and antibacterial activities and can also inhibit angiogenesis *in vivo* and *in vitro* ^30–35^. Chalcone analogues have attracted a great deal of interest due to their synthetic and biological importance in medicinal chemistry^36^. Recently, in a screen for LRAs that can reverse HIV latency, we have come upon a series of 2′-hydroxy-5-adamantyl-chalcones that display such an activity. One of them, called Amt-87, dose-dependently activates HIV transcription while displaying only mild cytotoxicity. It can also synergize with traditional LRAs such as prostratin and JQ1 to reactivate latent proviruses. Mechanistically, Amt-87 is shown to increase the phosphorylation of the CDK9 T-loop at position Thr186, dissociate P-TEFb from 7SK snRNP, and promote the assembly of the Tat-SEC complex on the HIV-1 LTR. Together, these data have revealed chalcones as a promising category of compounds that should be further explored to identify effective LRAs for targeted reversal of HIV latency.

## Results

### Identification of Amt-87 as a HIV latency-reversal agent (LRA)

To identify LRAs that can effectively reverse HIV latency, a systematic screen for compounds or natural products present in a library established in the School of Pharmaceutical Sciences at Xiamen University was conducted. This library contained chemically synthesized compounds as well as purified native products derived from traditional Chinese medicinal herbs, marine microbial secondary metabolites, and symbiotic bacteria secondary metabolites^37^. For detection of latency reversal, we employed the Jurkat T cell line-based J-Lat A2 system, which contains an integrated HIV-1 5′-LTR-Tat-Flag-iRES-EGFP-3′-LTR expression cassette^38^. This cassette is normally silent but produces the enhanced green fluorescent protein (GFP) upon activation, which can be detected by flow cytometry (FACS).

Among a family of structurally related chalcone derivatives, we found that significant induction of GFP expression in J-Lat A2 cells, i.e. reversal of HIV-1 latency, was caused by members **1a**, **1d**, **1h**, and **1l** (Table 1). The general method for the synthesis of 2′-hydroxy-chalcone amide derivatives (1a–1p) is outlined in Supplemental Information (Supplementary Fig. S1). Unfortunately, all four also markedly reduced cell viability (Table 2). To decrease the toxicity of these compounds and at the same time preserve their latency-reversing potential, we generated another two series of chalcone derivatives with functional group adamant (Ad) at the C5-position of the benzene ring system (**3a**–**3d** and **4a**–**4d**, see Table 1). Among these new derivatives, compound **3d** [(E)-3-(5-(adamantan-1-yl)-2,4-bis(methoxymethoxy) phenyl)-1-(2-hydroxy-5-methylphenyl)prop-2-en-1-one], called Amt-87 henceforth, effectively induced GFP production in J-Lat A2 cells at 50 and 100 μg/mL, while displaying significantly diminished cell toxicity at these concentrations compared to compounds in the other series (Table 2).

**Table 1:** Reactivation of latent HIV-1 gene expression by the indicated chalconoids in J-Lat A2 cells.

	
**Compound**	**Structures**		**% GFP(+) cells (50 μg/mL)**	**Compound**	**Structures**		**% GFP(+) cells (50 μg/mL)**
	R′	R			**R**′	**R**	
DMSO			0.7 ± 0.1	**1o**	2′-OH-4′-CH_3_	3-CONHCH_2_CH_2_-3′-Thi	3.1 ± 1.3
Prostratin (2.5 μm)			65.3 ± 5.4	**1p**	2′-OH-4′-CH_3_	3-CONHCH_2_CH_2_Ph	1.8 ± 0.3
**1a**	2′-OH-5′-CH_3_	4-CONHCH_2_CH_2_-3′-Thi	10.3 ± 2.0	**2a**	2′-OH	2-OH	2.0 ± 0.2
**1b**	2′-OH-5′-CH_3_	4-CONHPh	4.0 ± 1.5	**2b**	2′-OH	4-OH	1.2 ± 0.1
**1c**	2′-OH-5′-CH_3_	4-CONHCH_2_Ph	—	**2c**	2′4′-OH	4-OH	1.3 ± 0.2
**1d**	2′-OH-5′-CH_3_	4-CONH(CH_2_)_2_Ph	14.3 ± 3.3	**2d**	2′-OH-5′-CH_3_	4-OH	0.7 ± 0.2
**1e**	2′-OH-5′-CH_3_	4-CONH(CH_2_)_3_Ph	—	**2e**	4′-OH	2-OH-6-OCH_3_-3-prenyl	1.0 ± 0.2
**1f**	2′-OH-5′-CH_3_	4-CONH(CH_2_)_4_Ph	4.2 ± 1.1	**3a**	2′-OH	4-MOM-5-Ad	—
**1g**	2′-OH-5′-CH_3_	4-CONHCH_2_-1-Nap	2.6 ± 1.0	**3b**	2′-OH-5′-Cl	4-MOM-5-Ad	—
**1h**	2′-OH-5′-CH_3_	4-CONHPh-2′-OCH_3_	20.7 ± 3.8	**3c**	2′-OH-5′-CH_3_	4-MOM-5-Ad	3.9 ± 1.0
**1i**	2′-OH-5′-CH_3_	4-CONHC(CH_3_)_3_	—	**3d (Amt-87)**	2′-OH-5′-CH_3_	2,4-diMOM-5-Ad	4.9 ± 1.6
**1j**	2′-OH-5′-CH_3_	4-CONH-cyclo-C_4_H_7_	—	**3e**	5′-CH_3_	2,4-diMOM-5-Ad	—
**1k**	2′-OH-5′-CH_3_	4-CONH(CH_2_)_5_CH_3_	4.1 ± 1.2	**4a**	2′-OH	4-OH-5-Ad	—
**1l**	2′-OH-5′-CH_3_	4-CONH(CH_2_)_7_CH_3_	6.5 ± 1.4	**4b**	2′-OH-5′-Cl	4-OH-5-Ad	—
**1m**	2′-OH-5′-CH_3_	4-CONH-cyclo-C_8_H_15_	—	**4c**	2′-OH-5′-CH_3_	4-OH-5-Ad	3.5 ± 0.7
**1n**	2′-OH-5′-CH_3_	4-CONH-1-Ad	1.5 ± 0.2	**4d**	2′-OH-5′-CH_3_	2,4-OH-5-Ad	—

J-Lat A2 cells were treated for 24 hr with the empty vehicle DMSO, prostratin (2.5 μM) or the various chalconoids (50 μg/mL) of the indicated structures. Levels of HIV latency reversal were evaluated by FACS analysis to measure the percentage of cells that displayed the HIV LTR-driven GFP production. Data are presented as mean ± SD from at least three independent experiments performed in triplicates.

**Table 2:** Viability and GFP production in J-Lat A2 cells treated with the indicated chalconoids.

	
**Compound**	**R″**	**% GFP(+) cells (μg/mL)**			**% Cell viability (μg/mL)**		
		25	50	100	25	50	100
**1a**	4-CONHCH_2_CH_2_-3′-Thi	6.6 ± 0.7	9.2 ± 2.3	8.3 ± 1.6	67.1 ± 6.8	54.3 ± 5.4	41.8 ± 4.2
**1b**	4-CONHPh	3.1 ± 0.6	3.6 ± 1.2	3.0 ± 0.8	31.5 ± 4.4	15.7 ± 3.1	7.9 ± 2.2
**1d**	4-CONH(CH_2_)_2_Ph	13.7 ± 1.6	15.1 ± 2.4	15.7 ± 2.1	36.9 ± 3.9	35.0 ± 2.6	28.9 ± 3.7
**1f**	4-CONH(CH_2_)_4_Ph	2.5 ± 0.7	4.0 ± 1.3	7.2 ± 1.3	68.8 ± 3.1	65.2 ± 4.2	58.5 ± 4.5
**1g**	4-CONHCH_2_-1-Nap	10.3 ± 2.0	2.8 ± 1.0	1.8 ± 0.8	37.2 ± 6.2	27.9 ± 3.2	9.5 ± 1.5
**1h**	4-CONH-Ph-2′-OCH_3_	18.3 ± 1.3	22.5 ± 3.4	33.7 ± 3.6	49.3 ± 5.3	42.7 ± 4.0	12.3 ± 2.1
**1k**	4-CONH(CH_2_)_5_CH_3_	3.7 ± 1.2	3.9 ± 1.1	3.1 ± 1.0	67.6 ± 3.2	63.2 ± 4.2	53.3 ± 3.6
**1l**	4-CONH(CH_2_)_7_CH_3_	7.1 ± 1.7	8.4 ± 1.4	6.7 ± 1.5	24.7 ± 2.3	24.3 ± 2.7	19.5 ± 2.1
**1o**	3-CONHCH_2_CH_2_-3′-Thi	0.8 ± 0.1	3.8 ± 1.2	0.7 ± 0.1	36.4 ± 2.3	34.8 ± 2.7	37.3 ± 3.4
**1p**	3-CONH(CH_2_)_2_Ph	0.9 ± 0.1	2.1 ± 0.2	0.6 ± 0.1	42.0 ± 4.4	31.1 ± 2.0	31.2 ± 3.2
**3c**	4-MOM-5-Ad	0.7 ± 0.1	4.3 ± 1.1	13.3 ± 1.8	95.3 ± 3.5	92.5 ± 4.3	68.4 ± 4.2
**3d (Amt-87)**	2,4-diMOM-5-Ad	0.8 ± 0.1	5.9 ± 1.5	38.0 ± 4.2	96.3 ± 3.8	91.5 ± 3.4	72.9 ± 4.9
**4c**	4-OH-5-Ad	0.7 ± 0.1	3.8 ± 0.7	11.2 ± 2.0	86.4 ± 3.7	68.9 ± 6.3	45.4 ± 5.1

J-Lat A2 cells were treated with the indicated compounds at 25, 50 and 100 μg/mL concentrations for 24 hr and the percentage of GFP(+) cells and cell viability were evaluated by FACS and Cell viability analysis, respectively. Data are presented as mean ± SD from at least three independent experiments that were performed in triplicates.

**Figure 1:**
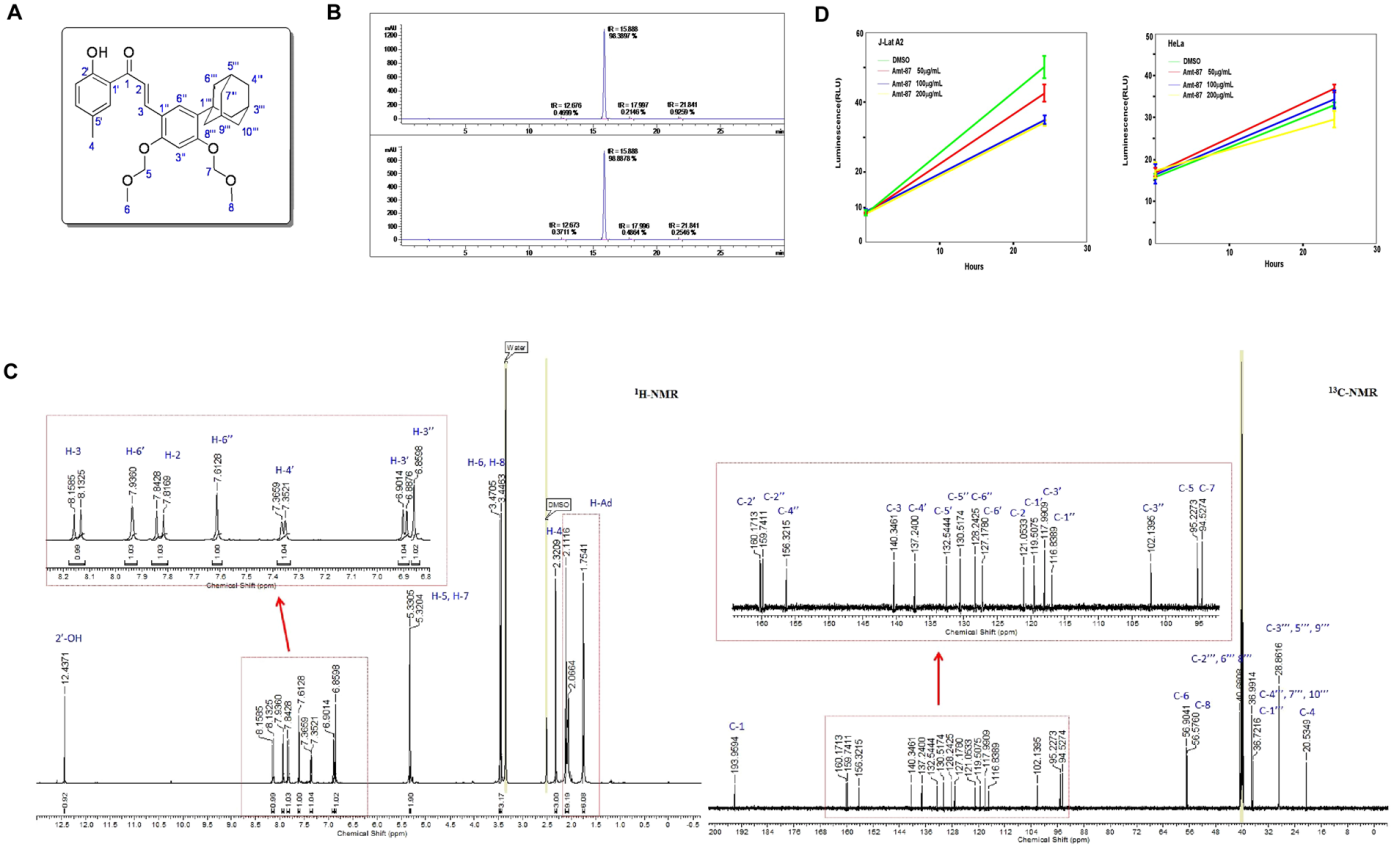
Purity analysis, structure confirmation and effect on cell viability of compound Amt-87. (**A**) The chemical structure of Amt-87 showing atom numbering. (**B**) HPLC-DAD analysis of Amt-87 performed at 254 nm (up) and 280 nm (down) is presented. The purity of Amt-87 is estimated to be above 98%. (**C**) The ^1^H-NMR (left) and ^13^C-NMR (right) spectra of Amt-87 are shown. The peaks in the spectra are identified and assigned to the structure. (**D**) The impact of Amt-87 on viability of J-Lat A2 and HeLa cells was measured at the indicated time points after the commencement of treatment by using the Promega CellTiter-Glo® Luminescent Cell Viability Assay kit. The error bars represent mean ± SD from three independent experiments.

The chemical structure of Amt-87 is shown in Fig. 1A and its purity was confirmed by High-Performance Liquid Chromatography with Diode-Array Detection (HPLC-DAD) performed at 254 nm (up) and 280 nm (down) in Fig. 1B, as well as the examination by ^1^H-NMR (left) and^13^C-NMR (right) in Fig. 1C. The general chemistry of the synthesis of Amt-87 is outlined in Supplemental Information (Supplementary Fig. S2). To further determine whether Amt-87 may affect cell growth, we used the Promega CellTiter-Glo® Luminescent Cell Viability Assay kit to measure the viability of both J-Lat A2 and HeLa cells that were treated with Amt-87 at concentrations of 50, 100 and 200 μg/mL for 24 hours. As indicated in Fig. 1D, these concentrations had no or only a very minor effect on the viability of HeLa cells when compared with the empty vehicle DMSO. For J-Lat A2, although these cells were more sensitive to Amt-87, treatment with 50 ug/ml of the drug, which was used in most latency-reversal experiments described below, had a relatively mild effect on cell viability.

Based on the above results showing that among the entire collection of chalcone derivatives, Amt-87 displayed the most optimal properties in terms of its efficient latency-reversing activity and relatively mild cytotoxicity, it was thus selected for further mechanistic study as outlined below.

### Amt-87 reactivates latent HIV at the transcriptional level

**Figure 2:**
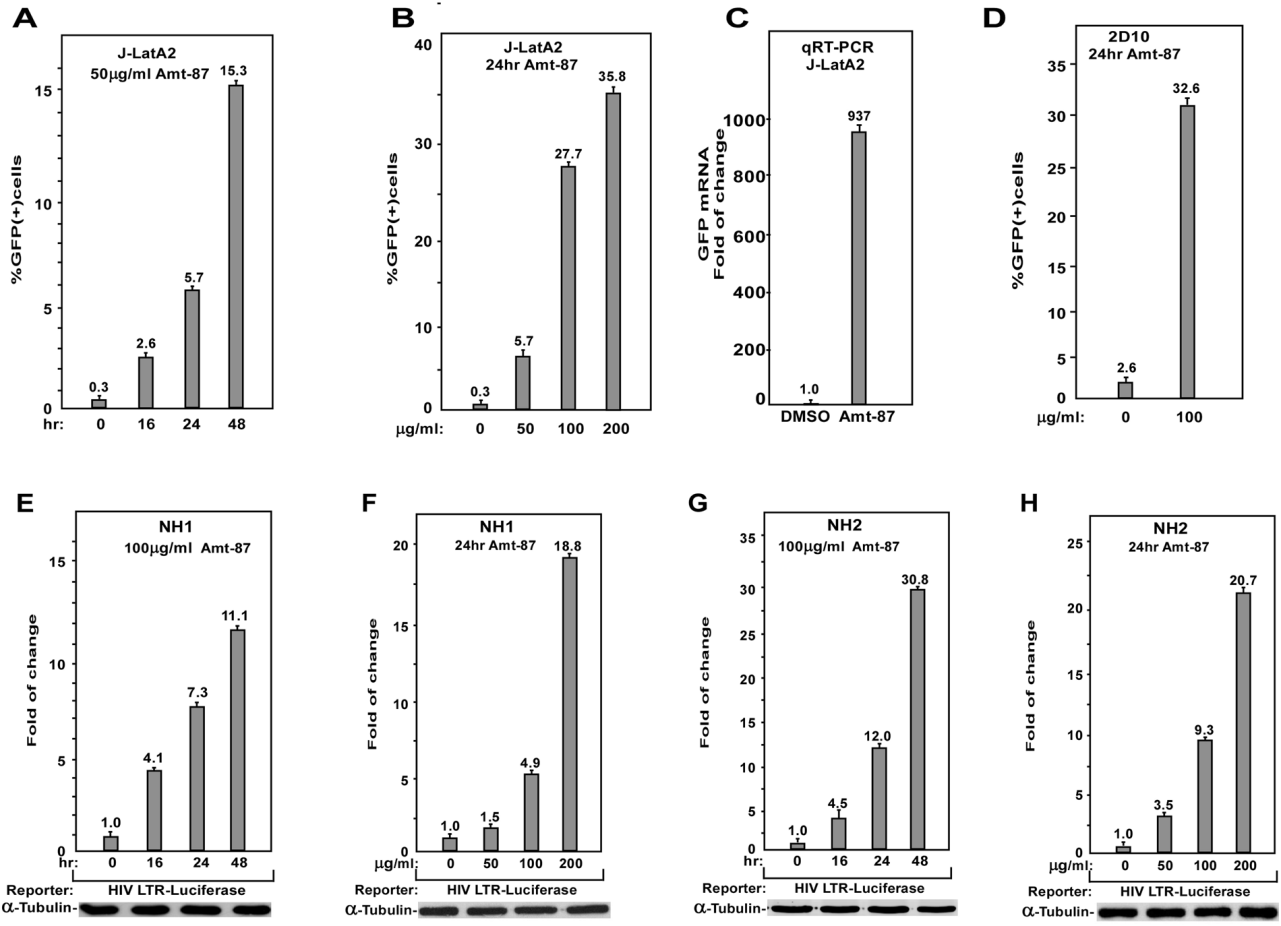
Amt-87 promotes reversal of HIV latency in a time- and does-dependent manner. (**A**,**B**) J-LatA2 cells were treated with 50 μg/ml of Amt-87 for the indicated time periods (**A**) or the indicated concentrations of the drug for 24 hr (**B**). The percentages of cells expressing GFP were measured by FACS and plotted. (**C**) J-LatA2 cells were treated with DMSO or 200 μg/ml Amt-87 for 24 hr. The GFP mRNA levels were measured by qRT-PCR and normalized to those of GAPDH and plotted, with the value in DMSO-treated cells adjusted to 1. (**D**) Jurkat 2D10 cells were treated with 100 μg/ml of Amt-87 or DMSO for 24 hr and the expression of GFP was analyzed by FACS and plotted. (**E**,**F**) The HeLa-based NH1 cells containing an integrated HIV LTR-luciferase reporter gene were treated with 100 μg/ml of Amt-87 for the indicated time periods (**E**) or 24 hr with the indicated concentrations of the drug (**F**). (**G**,**H**) The NH2 cells expressing Tat were treated with 100 μg/ml of Amt-87 for the indicated time periods (**G**) or 24 hr with in indicated concentrations of the drug (**H**). Whole cell extracts were prepared and examined for the luciferase activities. The error bars in all panels represent mean ± SD based on at least three independent experiments.

When J-Lat A2 cells were treated with different amounts of Amt-87 and for various time periods, the percentage of GFP-positive cells increased in a dose- and time-dependent manner (Fig. 2A,B). In addition, the treatment also significantly augmented the mean fluorescence intensity (MFI) of the GFP-positive cells (Supplementary Fig. S3). To confirm that the enhanced GFP signal detected by FACS was due to the increased production of GFP mRNA, quantitative RT-PCR (qRT-PCR) with primers that hybridize to a distal portion of the GFP gene was performed. The result showed that Amt-87 indeed reactivated the HIV provirus through stimulating the LTR-driven mRNA production (Fig. 2C), suggesting that the activation occurred at the transcriptional level.

The 2D10 cell line is another Jurkat-based post-integrative latency model that harbors almost the complete HIV genome except for the *nef* gene that is replaced by the GFP-coding sequence^39^. Importantly, the stimulatory effect of Amt-87 to reactivate latent HIV was also confirmed in this system (Fig. 2D). Finally, to ensure that the Amt-87-induced HIV transactivation was dependent on the viral 5′-LTR but not any other unrelated viral or non-viral sequences in the integrated expression cassette, we tested the effect of Amt-87 on expression of an integrated luciferase reporter gene that is driven solely by the HIV-1 5′-LTR in a pair of HeLa-based isogenic cells lines, NH1 and NH2, both of which contain an integrated HIV-1 5′ -LTR-luciferase reporter gene but only the latter stably expresses HIV-1 Tat^40, 41^. When these cells were treated with different amounts of Amt-87 and for various time periods as indicated in Fig. 2E–H, Amt-87 displayed a stimulatory effect on both Tat-independent and –dependent HIV-1 transcription in a dose- and time-dependent manner.

### Amt-87 synergizes with conventional LRAs prostratin and JQ1 to reactivate latent HIV

**Figure 3:**
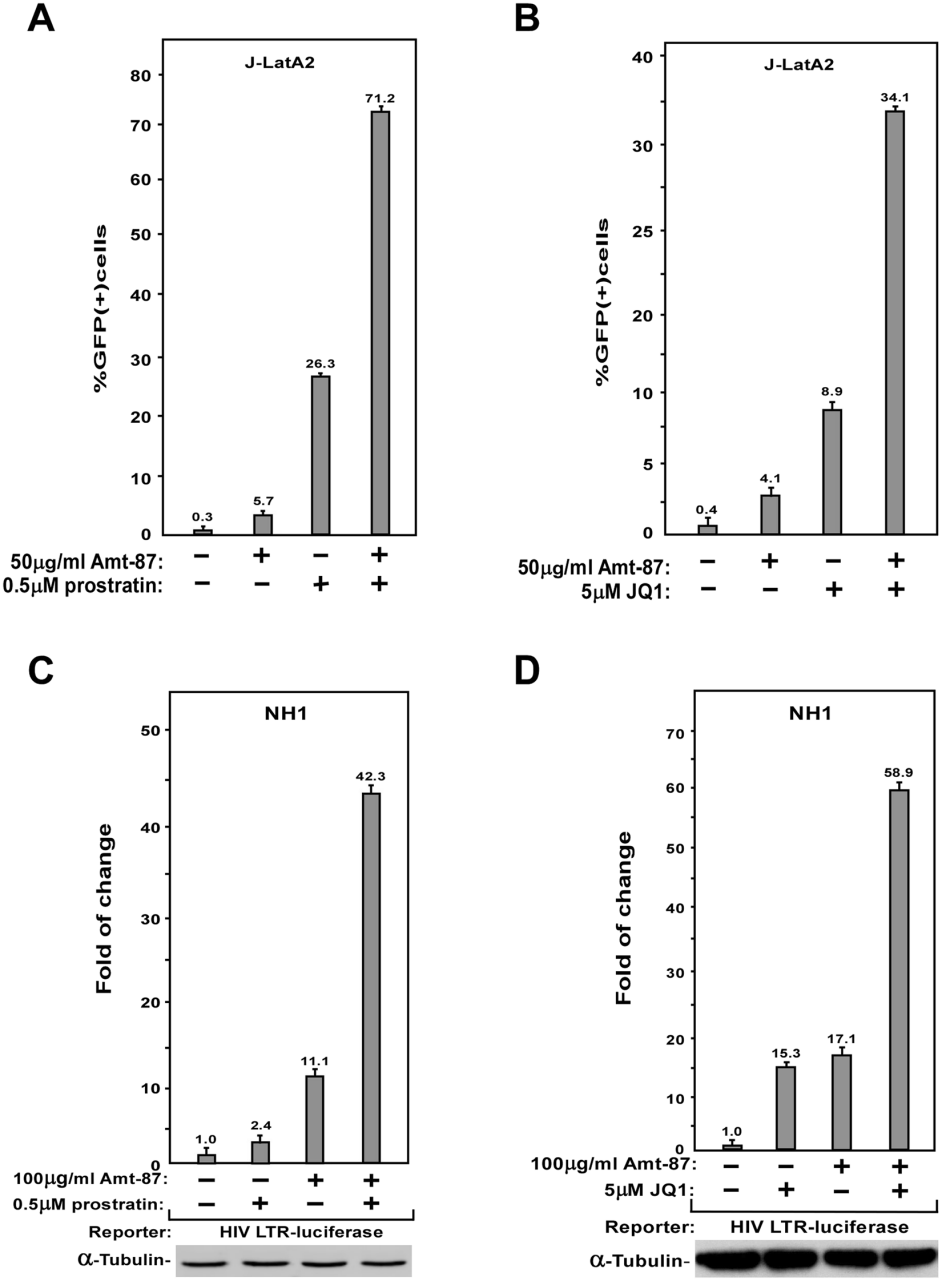
Amt-87 synergizes with prostratin and JQ1 to reverse HIV latency and reactivate viral transcription. (**A**,**B**) J-LatA2 cells were pretreated with prostratin (**A**) or JQ1 (**B**) for 2 hr, incubated with the indicated concentrations of Amt-87 for 24 hr, and then analyzed by FACS to determine the percentages of GFP(+) cells. (**C**,**D**) NH1 cells were pretreated with prostratin (**A**) or JQ1 (**B**) for 2hr, incubated with the indicated concentrations of Amt-87 for 24 hr, and then analyzed by FACS to determine the percentages of GFP(+) cells. Whole cell extracts (WCE) were examined for luciferase activities. The error bars in all panels represent mean ± SD from three independent experiments.

Previous studies have identified several classes of chemical compounds that can reactivate latent HIV. For example, prostratin is the prototype of protein kinase C (PKC) agonist that acts through and NF-κB pathway to promote the recruitment of RNA Pol II to the HIV-1 LTR^42^. On the other hand, JQ1, a BET bromodomain inhibitor, induces the reversal of HIV latency through antagonizing the inhibitory action of Brd4 on Tat-dependent activation of HIV transcriptional elongation^43^. To investigate how Amt-87 might work together with these two known conventional LRAs, J-Lat A2 cells were treated with Amt-87 alone or in combination with prostratin (Fig. 3A) or JQ1 (Fig. 3B). The data indicate that the combined treatments produced a much greater percentage of GFP(+) cells than the simple sum of the effects produced by either compound alone. Notably, the synergism displayed by Amt-87 together with prostratin or JQ1 was also recapitulated in NH1 cells containing the integrated HIV-1 LTR-driven luciferase reporter gene (Fig. 3C,D).

### CDK9 kinase activity is required for Amt-87 to reverse HIV latency

**Figure 4:**
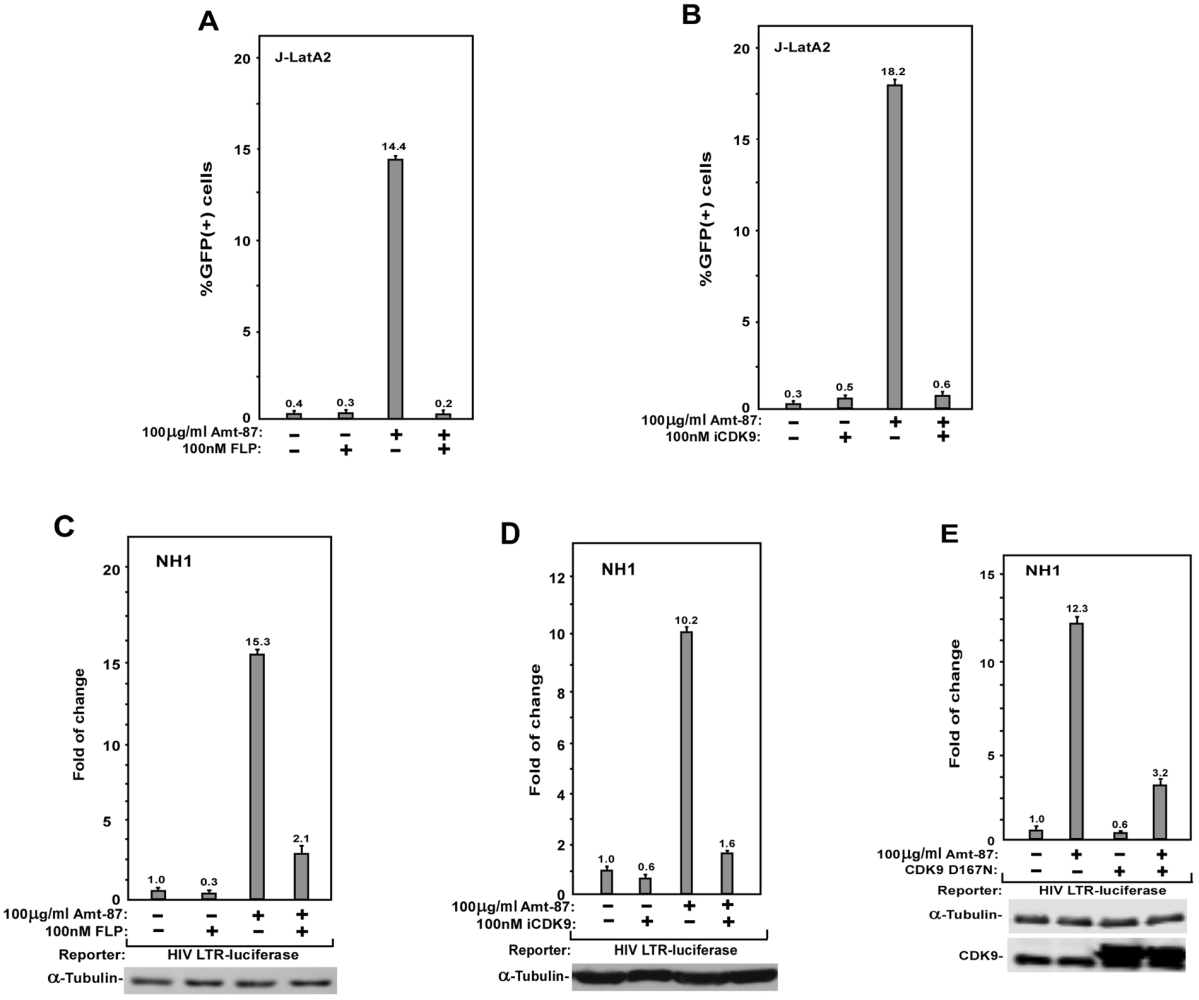
The kinase activity CDK9 is essential for Amt-87 to reactivate latent HIV-1 transcription. (**A**,**B**) J-LatA2 cells were pre-treated with flavopiridol (**A**) or iCDK9 (**B**) for 2 hr and then treated with Amt-87 for 24 hr at the indicated concentrations. The percentages of GFP(+) cells were determined by FACS and plotted. (**C**,**D**) NH1 cells were pre-treated with flavopiridol (**C**) or iCDK9 (**D**) and then treated with Amt-87 at the indicated concentrations for 24 hr. Whole cell extracts (WCE) were examined for luciferase activities. (**E**) NH1 cells were transfected with the plasmid expressing CDK9 mutant D167N and then treated with Amt-87 for 24 hr at the indicated concentration. WCE were examined for luciferase activities. The error bars in all panels represent mean ± SD from three independent experiments.

P-TEFb, which is composed of CDK9 and CycT1, plays a key role in HIV transcriptional activation, and its sequestration in the inactive 7SK snRNP has been proposed to contribute to viral latency^15^. To determine whether the kinase activity of CDK9 is required for Amt-87 to induce latency reversal, J-LatA2 cells were pre-treated with the CDK9 inhibitor flavopiridol^44^ or the more selective CDK9 inhibitor iCDK9^45^ before the incubation with Amt-87. As shown in Fig. 4A,B, these two drugs completely blocked the reactivation of HIV latency induced by Amt-87.

The dependence on CDK9 kinase activity for Amt-87′s stimulatory effect was also observed in NH1 cells, where the pre-treatment with not only flavopiridol (Fig. 4C) but also iCDK9 (Fig. 4D) markedly suppressed the ability of Amt-87 to activate the LTR-driven luciferase expression. Finally, similar to these two small molecule inhibitors of CDK9, D167N, a dominant negative form of CDK9^46^, was also able to prevent Amt-87 from activating the HIV-1 LTR once overexpressed in NH1 cells (Fig. 4E). Taken together, these dates indicate that the reversal of HIV latency by Amt-87 depended on CDK9’s kinase activity.

### Amt-87 promotes CDK9 T-loop phosphorylation

**Figure 5:**
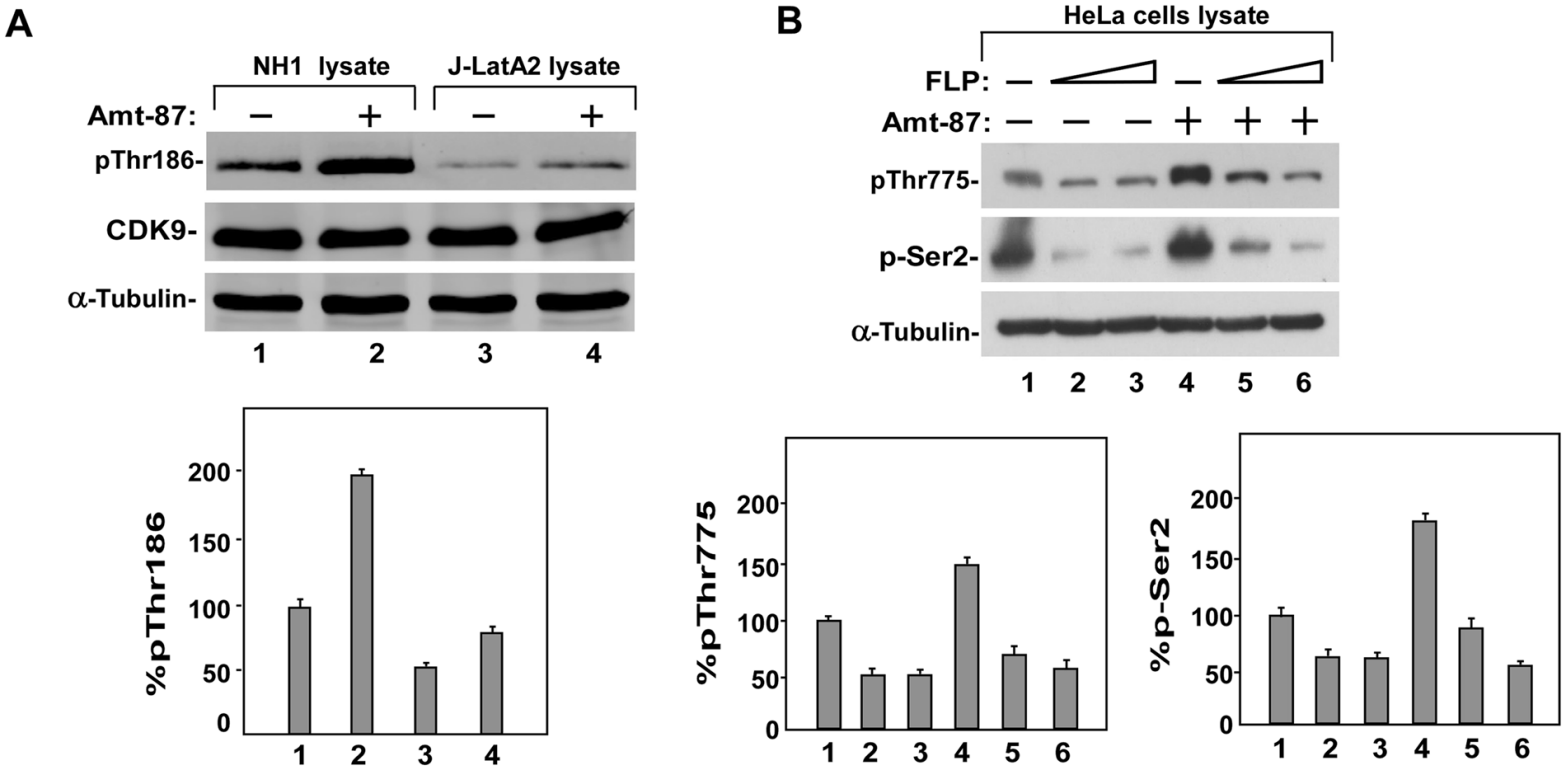
Amt-87 promotes phosphorylation of CDK9 T-loop at Thr186. (**A**) Whole cell lysates derived from NH1 and J-Lat A2 cells, which were treated with (+) or without (−) Amt-87 for 24 hr, were analyzed by Western blotting to detect the levels of total CDK9 and CDK9 phosphorylated on Thr186 (pT186). The levels of pT186 were quantified by densitometry, normalized to those of total CDK9, and shown below the blots, with the level of pT186 in untreated cells (lanes 1 & 3) artificially set at 100%. (**B**) HeLa cells were pre-treated with flavopiridol (FLP) for 2 hr and then treated with Amt-87 for 24 hr. The levels of Pol II CTD phosphorylated on Ser2 (pSer2) and Spt5 phosphorylated on Thr775 (pT775) in whole cell lysates were examined by Western blotting and quantified by densitometry as in A and shown in below the blots.

Given the above demonstrations that the stimulatory effect of Amt-87 depends on the active P-TEFb and the fact that the activation of P-TEFb during transcription elongation requires the phosphorylation of CDK9 on Thr186 at the T-loop^47, 48^, we next examined the phosphorylation status of Thr186 in NH1 and J-LatA2 cells after the treatment with Amt-87. Western analysis employing the phospho-Thr186 (pThr186)-specific antibody^47^, revealed that the level of phosphorylation on the CDK9 T-loop significantly increased upon the treatment in both cell lines (Fig. 5A).

In order to release Pol II from promoter-proximal pausing, active P-TEFb is known to phosphorylate the Pol II CTD on Ser2 and the Spt5 subunit of DSIF on Thr775^45^. We next performed Western blotting analysis using phospho-specific antibodies to examine the effect of Amt-87 on the phosphorylation status at these two positions. As shown in Fig. 5B, Amt-87 increased the levels of both pSer2 and pThr775, and this increase was abolished when CDK9 activity was inhibited by flavopiridol. Collectively, these data demonstrate that Amt-87 reactivated HIV latency through enhancing the phosphorylation of the CDK9 T-loop at Thr186, which turned on P-TEFb’s kinase activity to phosphorylate Pol II CTD on Ser2 and DSIF Spt5 on Thr775 to activate HIV transcriptional elongation.

### Amt-87 induces P-TEFb dissociation from 7SK snRNP and promotes Tat-SEC formation on HIV-1 LTR

**Figure 6:**
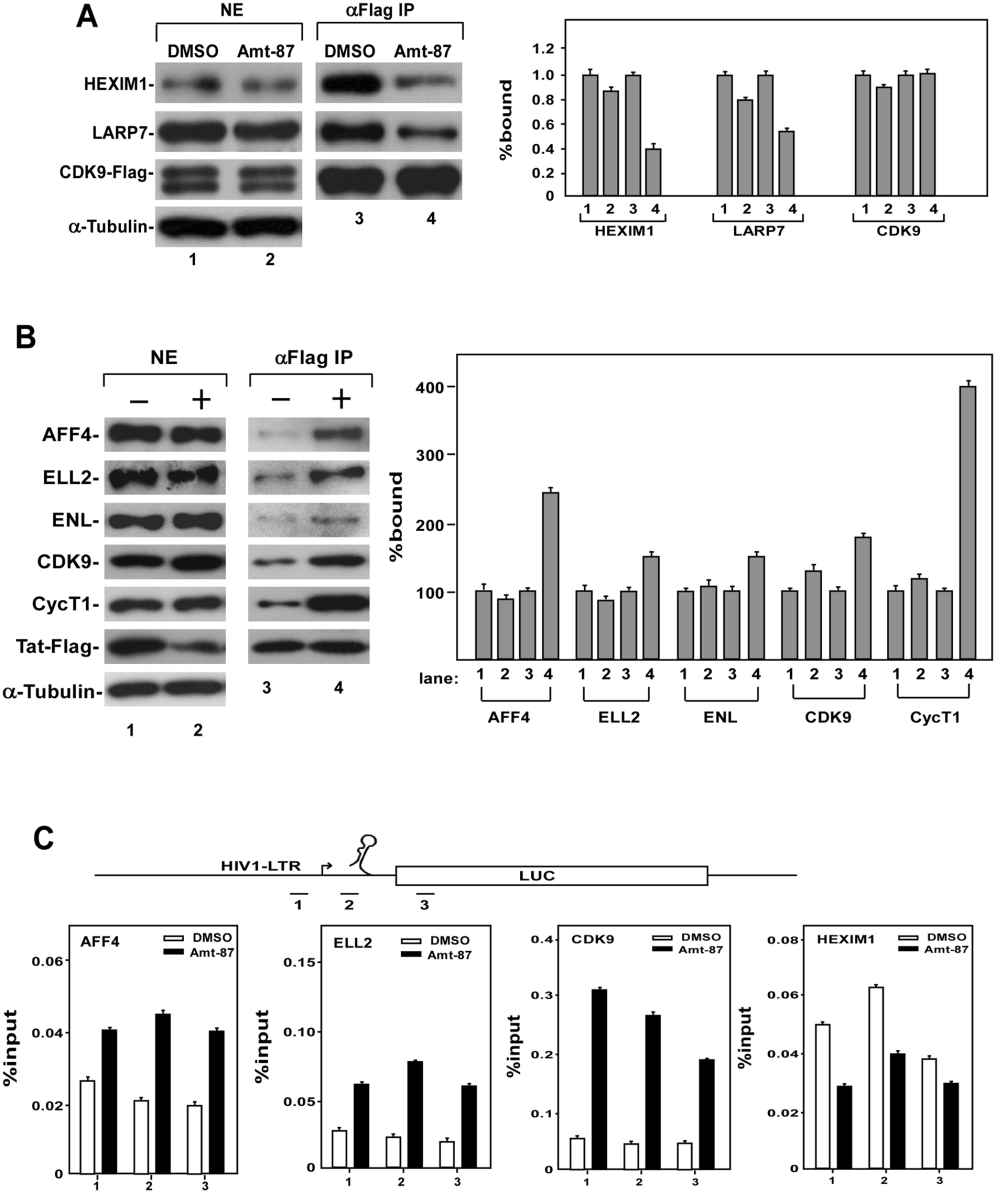
Amt-87 dissociates P-TEFb from 7SK snRNP and promotes Tat-SEC formation at the HIV-1 promoter. (**A**) The HeLa-based F1C2 cells stably expressing the Flag-tagged CDK9 were treated with Amt-87 or DMSO for 24 hr. Nuclear extracts (NE) and anti-Flag immunoprecipitates (IP) derived from NE were analyzed by Western blotting for the indicated proteins and the results were quantified and shown on the right, with the numbers in lanes 1 and 3 set to 1. (**B**) NH1 cells transfected with the Tat-Flag-expressing plasmid were treated with Amt-87 (+) or DMSO (−) for 24 hr. NEs and anti-Flag IP derived from NE were analyzed by Western blotting for the indicated proteins. (**C**) Upon transfection with the plasmid expressing Tat-Flag for 24 hr, NH1 cells were treated with Amt-87 or DMSO for 24 hr and then subjected to ChIP-qPCR analysis to determine the occupancy of the indicated factors at three different locations (1–3) at and around the HIV-1 promoter region (diagramed on top). The ChIP signals were normalized to those of input and plotted, with the error bars indicating mean ± SD from three independent experiments.

In latently infected cells, most of the cellular P-TEFb are sequestered in the inactive 7SK snRNP, and the release of P-TEFb from this complex is essential for latency reactivation^4^. Since Amt-87 has been shown above to activate P-TEFb by causing Thr186 phosphorylation on CDK9, we asked whether this drug is capable of inducing P-TEFb’s dissociation from 7SK snRNP. To answer this question, anti-Flag immunoprecipitation was performed in the HeLa-based F1C2 cells, which stably express the Flag-tagged CDK9 (CDK9-Flag)^41^, to examine the association of CDK9 with two signature 7SK snRNP components HEXIM1 and LARP7, which inhibits the CDK9 kinase activity and binds to the 3′ end of 7SK snRNA, respectively^49^. Using DMSO as a negative control, we found that Amt-87 markedly reduced the amounts of HEXIM1 and LARP7 bound to the immunoprecipitated CDK9-Flag (Fig. 6A), indicating a disruption of the 7SK snRNP in Amt-87-treated cells.

Given the ability of Amt-87 to directly disrupt 7SK snRNP and activate P-TEFb, we next investigated whether the released P-TEFb can be recruited by HIV-1 Tat into a SEC to form the Tat-SEC complex for activation of HIV transcription and latency reversal. Indeed, anti-Flag immunoprecipitates derived from the HeLa-based NH1 cells expressing Tat-Flag showed increased associations of the signature SEC components AFF4, ELL2, ENL, CDK9 and CycT1 with Tat-Flag upon the exposure to Amt-87 (Fig. 6B), suggesting an enhanced interaction between Tat and the SEC in Amt-87-treated cells.

To determine whether the enhanced Tat-SEC formation promoted by Amt-87 would result in an increased occupancy of the SEC at the HIV-1 promoter region, we performed the ChIP-qPCR analysis to examine the levels of CDK9, AFF4, ELL2 as well as the key 7SK snRNP subunit HEXIM1 at three separate locations along the integrated HIV-1 LTR-luciferase reporter gene. Indeed, compared to DMSO, Amt-87 increased the bindings of SEC components ELL2 and AFF4 as well as CDK9 at and around the HIV-1 promoter region, and decreased the binding of HEXIM1 especially at the HIV-1 promoter (Fig. 6C). These results are consistent with the notion that P-TEFb is transferred from the 7SK snRNP to the SEC in the presence of Amt-87. Taken together, these data support the model that by promoting T-loop phosphorylation in CDK9 and release of P-TEFb from 7SK snRNP, Amt-87 can increase the active pool of P-TEFb in the nucleus for formation of more Tat-SEC at the HIV-1 LTR, which in turn activates HIV-1 transcription and reverses viral latency.

## Discussion

The data presented in the current study are consistent with the notion that Amt-87 promotes the reversal of HIV latency by activating P-TEFb as well as P-TEFb-dependent viral transcription. Surprisingly, Amt-87 is shown to use three different means to activate P-TEFb, namely the induction of phosphorylation on the T-loop of CDK9 at Thr186, the disruption of the inactive 7SK snRNP to release P-TEFb, and lastly the promotion of Tat-SEC interaction on the viral LTR.

The latter two events are likely interconnected and may even have a causative relationship, as the release of P-TEFb from 7SK snRNP is expected to increase the pool of free and active P-TEFb that can be assembled into the SEC and recruited by Tat to the viral promoter region. However, it is unclear whether the Amt-87-induced phosphorylation of CDK9 at Thr186 is mechanistically linked to the disruption of 7SK snRNP. Previously, we and others have shown that even though CDK9 existing in the 7SK snRNP lacks kinase activity, it is nonetheless already phosphorylated at Thr186^50, 51^, an event, by analogy to other structurally determined CDKs^52^, locks the T-loop in an open conformation to allow the access to the catalytic center by the kinase substrate, and thus makes CDK9 poised to become active once liberated from the 7SK snRNP.

What remains to be determined is whether the Amt-87-induced Thr186 phosphorylation contributes to or may even be responsible for the release of P-TEFb from 7SK snRNP. Another aspect of the Amt-87 action that is yet to be investigated concerns how the drug causes the phosphorylation of CDK9 and disruption of 7SK snRNP. Our preliminary data suggest that Amt-87 very likely triggers an unknown signaling pathway leading to these two events, as the incubation of the drug with purified P-TEFb and 7SK snRNP *in vitro* affected neither the T-loop phosphorylation nor 7SK snRNP integrity (Supplementary Figs S4 and S5).

Chalconoids, which exist widely in fruits, vegetables, spices and tea, have interested organic and medicinal chemists for many years^30^. This is because chalconoids and their derivatives are not only important in the biosynthesis of flavonoids as both precursors and products, they also possess numerous pharmacological activities such as antimicrobial, anti-inflammatory, antiviral, and anticancer effects^30^. Here, we report a novel function of the chalconoid derivative, Amt-87, in the reversal of HIV latency. Using two different Jurkat cell line-based latency models (J-Lat and 2D10) as well as the HIV-1 LTR-driven luciferase reporter system, we provide evidence that Amt-87 promotes the exit of HIV proviruses from latency by stimulating viral transcription. These observations indicate that chalconoids can be a promising category of compounds that should be further explored to identify effective LRAs that can be used alone or in combination with other drugs to induce efficient reversal of HIV latency.

## Materials and Methods

### Chemistry

All reagents and chalcones 2a–2e were purchased from commercial sources and were used without further purification unless otherwise indicated. Reactions, which required the use of anhydrous, inert atmosphere techniques, were carried out under an atmosphere of nitrogen. Column chromatography was executed with silica gel (60–120 or 230–400 mesh) using mixtures of ethyl acetate and hexane as eluants. Melting points were measured on a SGW X-4 micro-melting point spectrometer and were uncorrected. ^1^H-NMR and ^13^C-NMR spectra were obtained using a Bruker (Bruker Biospin, Zug, Switzerland) AV2 600 Ultrashield spectrometer at 600 and 150 MHz, respectively. Chemical shifts were given in parts per million (ppm) relative to tetramethylsilane (TMS) as an internal standard. Multiplicities were abbreviated as follows: single (s), doublet (d), doublet-doublet (dd), doublet-triplet (dt), triplet (t), triplet-triplet (tt), triplet-doublet (td), quartet (q), quartet-doublet (qd), quint (quin), sext (sxt), multiplet (m), and broad signal (br. s.). High-resolution mass spectral (HRMS) data were acquired using electrospray ionization (ESI) on a Q Exactive LC-MS/MS instrument (Thermo Fisher Scientific Inc., Waltham, MA, USA) with UV detection at 254 nm.

### Synthesis

The general method for the synthesis of 2′-hydroxy-chalcone amide derivatives (1a–1p) and 5-adamantyl-chalcones (3a–3e and 4a–4d) are outlined in Supplemental Information, with their structures confirmed by ^1^H-NMR, ^13^C-NMR, and HRMS (for details, see Supplemental Information).

### HPLC analysis of Amt-87

The purity of Amt-87 was assessed by HPLC with a COSMOSIL C18-MS-II column (250 mm × 4.6 mm i.d., 5 mm). The mobile phase used was acetonitrile (A) and water (B) in a linear gradient mode as follows: A from 80% to 100% and B from 20% to 0% during 0–20 min. The HPLC analysis was performed at a flow rate of 1 mL/min and monitored at 254 nm and 280 nm by the Agilent HPLC system.

### Biochemistry

#### Reagents, antibodies and cell lines

Prostratin and JQ1 were purchased from LC Laboratories and Cayman Chemical, respectively. Flavopindol (F3055) was purchased from Sigma. The anti-ENL (A302-267A) and anti-ELL2 (A302-505A) antibodies were purchased from Bethyl Laboratories. The antibodies against α-tubulin (T9026) and Flag (F3165) were obtained from Sigma. Antibodies recognizing CycT1 (SC-8128) and AFF4 (14662-1-AP) were purchased from Santa Cruz Biotechnology and Proteintech, respectively. Rabbit polyclonal antibodies against Brd4, HEXIM1, CDK9, LARP7, pThr186 of CDK9, pSer2 of RNA Pol II and pThr775 of Spt5 have been described previously^26, 47, 53^. Rabbit total IgG (I5006) used for ChIP was purchased from Sigma. All the cell lines used in this study were either purchased from American Type Culture Collection (ATCC, Manassas, VA) or described before^54^.

#### Screening by flow cytometry

The flow cytometry-based screening was performed in J-Lat A2 cells containing the integrated LTR-Tat-IRES-GFP-LTR cassette^38^ and 2D10 cells harboring the nearly complete HIV-1 genome except for the *nef* gene that is replaced by the GFP-coding sequence^39^. After incubation with the indicated concentrations of Amt-87 or 0.1% dimeththyl sulfoxide (DMSO) as a negative control, cells were harvested and washed twice in 1× phosphate buffered saline (PBS) and analyzed by flow cytometer on Epics Altra (BECKMAN COULTER) for the percentages of cells expressing GFP.

#### Luciferase assay

The luciferase assay was conducted in NH1 and NH2, a pair of HeLa-based isogenic cell lines, both of which contain an integrated HIV-1 LTR-luciferase reporter gene but only the latter stably expresses HIV-1 Tat^40, 41^. NH1 and NH2 cells were treated with different amounts of Amt-87 and for various time periods as indicated or DMSO as a negative control. Cell lysates were extracted and subjected to the measurement of luciferase activity using a kit from Promega.

#### Co-immunoprecipitation (co-IP)

The co-immunoprecipitation assay was performed as described^16^ with minor modifications. Briefly, to detect the dissociation of 7SK snRNP after drug treatment, nuclear extracts (NEs) were prepared from F1C2 cells, a HeLa-based cell line stably expressing Flag-tagged CDK9^53^. To detect the drug-induced change of the SEC, NH1 cells were transfected with 20 μg pRK5M-Tat-Flag. One day later, the cells were treated with 200 μg/ml Amt-87 or DMSO as a control for 24 hr and then subjected to the preparation of NEs. For anti-Flag IP, NEs were incubated with anti-Flag agarose beads (Sigma) for 2 hr at 4 μL at a ratio of 10 μL beads per 100 μL NEs. After extensive washing, the eluted proteins were analyzed by Western blotting with the indicated antibodies.

#### Chromatin immunoprecipitation (ChIP) assay

The assay was performed essentially as described^55^ with minor modifications. Briefly, NH1 cells containing a stably integrated HIV-1 LTR-luciferase reporter gene were transfected with the expression construct pRK5M-Tat-Flag. Twenty four hours later, the transfected cells were treated with Amt-87 (100 μg/ml) or DMSO for 24 hr. Cells were cross-linked with 1% formaldehyde for 10 min at room temperature, which was quenched by the addition of glycine to 0.125 M for 5 min. After washing in PBS, 1 × 10^7^ cells were re-suspended in 300 μL SDS lysis buffer (1% SDS, 10 mM EDTA, 50 mM Tris-HCl, pH 8.0) and fragmented (5 sec ON, 10 sec OFF, for a total of 20 cycles) using a Scientz JY92-IID sonicator. The lysates were diluted 10 times with the dilution buffer (0.01% SDS, 1.1% Triton X-100, 1.2 mM EDTA, 16.7 mM Tris-HCl, pH 8.1, and 167 mM NaCl), pre-cleared with Protein A or G beads (GE), and incubated with the various antibodies (5 μg anti-CDK9, 3 μg anti-HEXIM1, 1.5 μg anti-AFF4, 1.5 μg anti-ELL2, or 2 μg rabbit total IgG) overnight at 4 μL. After mixing with Protein A/G beads for 1 hr, the beads were washed three times with the washing buffer (0.1% SDS, 1% Triton X-100, 2 mM EDTA, 20 mM Tris-HCl, pH 8.1, and 150 mM NaCl) and then three times with the TE buffer (10 mM Tris-HCl, pH 8.1 and 1 mM EDTA). DNA was eluted from the beads with the elution buffer (1% SDS and 100 mM NaHCO_3_) and purified by phenol/chloroform extraction and ethanol precipitation. The final products were analyzed by real-time PCR with the primers listed below to amplify the integrated HIV LTR-luciferase gene. The PCR signals were normalized to the input DNA and shown as an average of three independent measurements. The sequences of the primers are:

Promoter (position 1) Forward: 5′ TCCGCTGGGGACTTTCCA

Promoter (position 1) Reverse: 5′ GTACAGGCAAAAAGCAGCTGC

TAR(position 2) Forward: 5′TGCTTTTTGCCTGTACTGGGT

TAR(position 2) Reverse: 5′CAGTACCGGAATGCCAAGCTT

Nascent (position 3) Forward: 5′ TCTGGCTAACTAGGGAACCCA

Nascent (position 3) Reverse: 5′ CGCCGGGCCTTTCTTTATGT

### Data Availability

All data generated or analyzed during this study are included in this published article and its Supplementary Information files.

## Electronic supplementary material

Supplemental information
