# Supplementary material for: A chalcone derivative reactivates latent HIV-1 transcription through activating P-TEFb and promoting Tat-SEC interaction on viral promoter
Source: Sci Rep. 2017 Sep 6;7:10657. doi: 10.1038/s41598-017-10728-w (PMC5587564; doi:10.1038/s41598-017-10728-w)
Supplement: Supplementary file 1 — Supplemental information [file 41598_2017_10728_MOESM1_ESM.pdf]

A chalcone derivative reactivates latent HIV-1 transcription through activating P-TEFb and promoting Tat-SEC interaction on viral promoter

Jun Wu[1¶], Ming-tao Ao[1¶], Rui Shao[1], Hui-ru Wang[1], Diao Yu[1], Mei-juan Fang[1], Xiang Gao[1], *Zhen Wu[1], *Qiang Zhou[2] & *Yu-hua Xue[1].

Synthesis

Compounds **1a-1p** were prepared according to a two-step procedure **(Supplementary Fig. S1)** previously reported in article[1,2]. Condensation of 1-(2-hydroxy-5-methylphenyl)ethanone with 3- or 4-carboxybenzaldehyde in the presence of alkali gave carboxylic acid derivatives **7a** and **7b**. Treatment of **7a** or **7b** in CH_2Cl_2 with various amines in the presence of 1-ethyl-3-(3-dimethylaminopropyl)carbodiimide (EDCI) and *N*-hydroxybenzotriazole (HOBt) afforded various amides **1a-1p** with yields ranged from 40 % to 60 %.

As shown in supplementary Fig. S2**,** the adamantyl-substituted benzaldehyde derivatives **9a** and **9b** were prepared by Friedel-Crafts alkylation of 4-hydroxybenzaldehyde and 2,4-dihydroxybenzaldehyde with adamantan-1-ol using sulfuric acid as catalyst, respectively. This was followed by phenol protection by treatment of **9a or 9b** with NaH in DMF at 0 ℃ followed by trapping with chloromethyl methyl ether (MOMCl) to afford **10a or 10b**. Chalcones **3a-3e** were then easily obtained in moderate yields (51%~53%) through Claisene Schmidt condensation of **10** and corresponding acetophenone under alkaline conditions[3]. For the synthesis of **4a-4d**, removal of methoxymethyl (MOM) protecting groups of **3a-3d** under acidic conditions gave the desired products[4]. All new chalcone derivatives were characterized by ^{1}H NMR, ^{13}CNMR, and HRMS.

General procedure for the synthesis of Chalcones. **Claisene Schmidt condensation.** To a solution of the corresponding aldehyde (1 equiv) and corresponding acetophenone (1 equiv) in EtOH (3 mL for 1 mmol of acetophenone) was added NaOH (5 equiv). The reaction mixture was stirred at room temperature for 24 h and neutralized with 10% HCl solution to form yellow precipitate. The yellow precipitate was filtered and washed with appropriate

amount of water. The crude product was purified by chromatography using hexane/EtOAc and recrystallized by MeOH to give the desired compounds.

(E)-4-(3-(2-hydroxy-5-methylphenyl)-3-oxoprop-1-en-1-yl)benzoic acid (7a)

Following the general procedure for the Claisen-Schmidt condensation, 1-(2-hydroxy-5-methylphenyl)ethanone **5** (1.1 mmol, 165.2 mg) and 4-carboxybenzaldehyde (1.0 mmol, 150.1 mg) were used to give **7a** as a yellow solid. Yield 53.8 %. ^{1}H NMR (600 MHz, C_5D_5N): δ 8.41 (d, J = 8. 2 Hz, 2H), 8.19 (d, J = 15.5 Hz, 1H) , 8.12 (d, J = 17.2 Hz, 1H), 8.07 (d, J = 1.0 Hz, 1H), 7.88 (d, J = 8.2 Hz, 2H), 7.31 (dd, J = 1.8, 8.4 Hz, 1H), 7.1l (d, J = 8.4 Hz, 1H), 2.18 (s, 3H). ^{13}C-NMR (150 MHz, C_5D_5N): δ 193.9, 168.3, 161.6, 143.9, 138.7, 137.7, 137.2, 130.5 (2C), 129.0 (2C), 128.3, 126.4 (2C), 120.5, 118.2, 20.0. ESI-HRMS (-): m/z [M-H]$^-$ calcd for $C_{17}H_{13}O_4^-$, 281.0819, found, 281.0816.

(E)-3-(3-(2-hydroxy-5-methylphenyl)-3-oxoprop-1-en-1-yl)benzoic acid (7b)

Following the general procedure for the Claisen-Schmidt condensation, 1-(2-hydroxy-5-methylphenyl)ethanone **5** (1.1 mmol, 165.2 mg) and 3-carboxybenzaldehyde (1.0 mmol, 150.1 mg) were used to give **7b** as a yellow solid. Yield 50.1 %. ^{1}H NMR (600 MHz, DMSO-d_6): δ 12.27 (s, 1H), 8.40 (s, 1H), 8.18 (d, J = 7.9 Hz, 1H), 8.08-8.14 (m, 2H), 8.03 (d, J = 7.7 Hz, 1H), 7.89 (d, J = 15.6 Hz, 1H), 7.62 (t, J = 7.7 Hz, 1H), 7.40 (dd, J = 1.7, 8.3 Hz, 1H), 6.92 (d, J = 8.3 Hz, 1H), 2.33 (s, 3H); ^{13}C NMR (150 MHz, DMSO-d_6): δ 193.9, 167.4, 160.3, 144.0, 137.8, 135.4, 133.5, 132.2, 131.8, 131.0, 130.2, 129.7, 128.5, 123.5, 120.8, 118.0, 20.4; ESI-HRMS (-): m/z [M-H]$^-$ calcd for $C_{17}H_{13}O_4^-$, 281.0819, found, 281.0814.

General procedure for synthesis of amide compounds 1a-1p. A mixture of **7** (**7a** or **7b**) (1 equiv), HOBt (1.2 equiv) and EDCI (1.2 equiv) was dissolved in CH_2Cl_2, and stirred for 30 min. The mixture was then added with appropriate amine (2.0 equiv), and stirred at the room temperature for 12 h. After completion of the reaction, the mixture was concentrated in vacuum to give the crude product. The crude product was purified by column chromatography with hexane/EtOAc, and recrystallized with EtOAc to afford pure products.

(E)-4-(3-(2-hydroxy-5-methylphenyl)-3-oxoprop-1-en-1-yl)-N-(2-(thiophen-3-yl)ethyl)benzamide (1a)

The title compound **1a** was obtained by the reaction of compound **7a** with 3-thiopheneethanamine following the general procedure. Yellow solid, yield 57.5 %, mp 182-183 ℃. ^{1}H NMR (600 MHz, $CDCl_3$): δ 12.46 (s, 1H), 7.79 (dd, *J* = 1.9, 15.5 Hz, 1H), 7.71 (d, *J* = 7.9 Hz, 2H), 7.57-7.63 (m, 4H), 7.25 (d, *J* = 8.4 Hz, 1H), 7.11 (d, *J* = 5.1 Hz, 1H), 6.90 (t, *J* = 4.2 Hz, 1H), 6.85 (dd, *J* = 1.5, 8.4 Hz, 1H), 6.81 (d, *J* = 2.4 Hz, 1H), 6.37 (br. s., 1H), 3.67 (q, *J* = 5.8 Hz, 2H), 3.09 (t, *J* = 6.4 Hz, 2H), 2.27 (s, 3H); ^{13}C NMR (150 MHz, $CDCl_3$): δ 192.3, 165.6, 160.5, 142.6, 140.1, 136.8, 136.5, 135.2, 128.3, 127.7 (2C), 127.1, 126.5 (2C), 126.2, 124.5, 123.1, 120.9, 118.5, 117.4, 40.4, 28.8, 19.6; ESI-HRMS (-): *m/z* $[M-H]^-$ calcd for $C_{23}H_{20}NO_3S^-$, 390.1169, found, 390.1155.

(E)-4-(3-(2-hydroxy-5-methylphenyl)-3-oxoprop-1-en-1-yl)-N-phenylbenzamide (1b)

The title compound **1b** was obtained by the reaction of compound **7a** with aniline following the general procedure. Yellow solid, yield 45.8 %, mp 203-205 ℃. ^{1}H NMR (600 MHz, DMSO-d_6): δ 12.27 (s, 1H), 10.35 (s, 1H), 8.16 (d, *J* = 15.6 Hz, 1H), 8.11 (d, *J* = 0.9 Hz, 1H), 8.07-8.10 (m, 2H), 8.03-8.06 (m, 2H), 7.89 (d, *J* = 15.6 Hz, 1H), 7.80 (d, *J* = 7.7 Hz, 2H), 7.41 (dd, *J* = 1.7, 8.4 Hz, 1H), 7.36-7.39 (m, 2H), 7.11-7.14 (m, 1H), 6.93 (d, *J* = 8.4 Hz, 1H), 2.34 (s, 3H); ^{13}C NMR (150 MHz, $CDCl_3$): δ 193.9, 165.3, 160.3, 143.8, 139.5, 137.9, 137.8, 137.0, 131.0, 129.5 (2C), 129.1 (2C), 128.7 (2C), 128.5, 124.3, 124.0, 120.9 (2C), 120.9, 118.0, 20.4; ESI-HRMS (-): *m/z* $[M-H]^-$ calcd for $C_{23}H_{18}NO_3^-$, 356.1292, found, 356.1297.

(E)-N-benzyl-4-(3-(2-hydroxy-5-methylphenyl)-3-oxoprop-1-en-1-yl)benzamide (1c)

The title compound **1c** was obtained by the reaction of compound **7a** with benzylamine following the general procedure. Yellow solid, yield 46.3 %, mp 170-172 ℃. ^{1}H NMR (600 MHz, $CDCl_3$): δ 12.47 (s, 1H), 7.83 (d, *J* = 15.4 Hz, 1H), 7.79 (d, *J* = 8.3 Hz, 2H), 7.66 (d, *J* = 8.3 Hz, 2H), 7.64 (d, *J* = 15.6 Hz, 1H), 7.61 (s, 1H), 7,30-7.31 (m, 4H), 7.24-7.27 (m, 2H), 6.88 (d, *J* = 8.4 Hz, 1H), 6.38 (br. s., 1H), 4.61 (d, *J* = 5.5 Hz, 2H), 2.29 (s, 3H); ^{13}C NMR (150 MHz, $CDCl_3$): δ 192.3, 165.4, 160.6, 142.6, 136.9, 136.8, 136.6, 135.0, 128.3, 127.9 (2C), 127.7 (2C), 127.1, 127.0 (2C), 126.8, 126.6 (2C), 121.0, 118.5, 117.4, 43.3, 19.6; ESI-HRMS (-): *m/z* $[M-H]^-$ calcd for $C_{24}H_{20}NO_3^-$, 370.1449, found, 370.1447.

(E)-4-(3-(2-hydroxy-5-methylphenyl)-3-oxoprop-1-en-1-yl)-N-phenethylbenzamide (1d)

The title compound **1d** was obtained by the reaction of compound **7a** with

2-phenylethanamine following the general procedure. Yellow solid, yield 52.3 %, mp 187-188 ℃. ^{1}H NMR (600 MHz, $CDCl_3$): δ 12.47 (s, 1H), 7.80 (d, J = 15.4 Hz, 1H), 7.67 (d, J = 8.3 Hz, 2H), 7.59-7.63 (m, 4H), 7.24-7.29 (m, 3H), 7.16-7.21 (m, 3H), 6.86 (d, J = 8.4 Hz, 1H), 6.17 (br. s., 1H), 3.67 (q, J = 6.8 Hz, 2H), 2.88 (t, J = 6.9 Hz, 2H), 2.28 (s, 3H); ^{13}C NMR (150 MHz, $CDCl_3$): δ 192.3, 165.6, 160.6, 142.6, 137.7, 136.8, 136.5, 135.3, 128.3, 127.8 (2C), 127.8(2C), 127.7 (2C), 127.0, 126.5 (2C), 125.7, 120.9, 118.5, 117.4, 40.2, 34.6, 19.6; ESI-HRMS (-): *m/z* [M-H]$^-$ calcd for $C_{25}H_{22}NO_3^-$, 384.1605, found, 384.1605.

(E)-4-(3-(2-hydroxy-5-methylphenyl)-3-oxoprop-1-en-1-yl)-N-(3-phenylpropyl)benzamide (1e)

The title compound **1e** was obtained by the reaction of compound **7a** with 3-phenylpropan-1-amine following the general procedure. Yellow solid, yield 46.8 %, mp 155-157 ℃. ^{1}H NMR (600 MHz, $CDCl_3$): δ 12.48 (s, 1H), 7.82 (d, J = 15.6 Hz, 1H), 7.62-7.66 (m, 4H), 7.61-7.62 (m, 2H), 7.22-7.28 (m, 3H), 7.13-7.17 (m, 3H), 6.88 (d, J = 8.4 Hz, 1H), 6.04 (br. s., 1H), 3.46 (q, J = 6.7 Hz, 2H), 2.68 (t, J = 7.5 Hz, 2H), 2.29 (s, 3H), 1.93 (quin, J = 7.2 Hz, 2H). ^{13}C NMR (150 MHz, $CDCl_3$): δ 192.3, 165.6, 160.6, 142.7, 140.4, 136.8, 136.4, 135.3, 128.3, 127.6 (2C), 127.6 (2C), 127.4 (2C), 127.1, 126.5 (2C), 125.1, 120.9, 118.5, 117.4, 39.0, 32.6, 30.0, 19.6. ESI-HRMS (-): *m/z* [M-H]$^-$ calcd for $C_{26}H_{24}NO_3^-$, 398.1762, found, 398.1732.

(E)-4-(3-(2-hydroxy-5-methylphenyl)-3-oxoprop-1-en-1-yl)-N-(4-phenylbutyl)benzamide (1f)

The title compound **1f** was obtained by the reaction of compound **7a** with 4-phenylbutan-1-amine following the general procedure. Yellow solid, yield 59.2 %, mp 177-179 ℃. ^{1}H NMR (600 MHz, $CDCl_3$): δ 12.48 (s, 1H), 7.81 (d, J = 15.6 Hz, 1H), 7.73 (d, J = 8.3 Hz, 2H), 7.59-7.64 (m, 4H), 7.25 (dd, J = 1.3, 8.4 Hz, 1H), 7.19-7.23 (m, 2H), 7.09-7.13 (m, 3H), 6.86 (d, J = 8.4 Hz, 1H), 6.15 (br. s., 1H), 3.41 (q, J = 6.8 Hz, 2H), 2.60 (t, J = 7.4 Hz, 2H), 2.28 (s, 3H), 1.64-1.68 (m, 2H), 1.59-1.61 (m, 2H); ^{13}C NMR (150 MHz, $CDCl_3$): δ 192.3, 165.6, 160.6, 142.7, 141.0, 136.8, 136.4, 135.4, 128.3, 127.7 (2C), 127.4 (2C), 127.4 (2C), 127.1, 126.5 (2C), 124.9, 120.8, 118.5, 117.4, 39.0, 34.5, 28.2, 27.7, 19.6; ESI-HRMS (-): *m/z* [M-H]$^-$ calcd for $C_{27}H_{26}NO_3^-$, 412.1918, found, 412.2001.

(E)-4-(3-(2-hydroxy-5-methylphenyl)-3-oxoprop-1-en-1-yl)-N-(naphthalen-2-ylmethyl)ben

zamide (1g)

The title compound **1g** was obtained by the reaction of compound **7a** with 1-naphthalenemethylamine following the general procedure. Yellow solid, yield 42.4 %, mp 197-199 ℃. ^{1}H NMR (600 MHz, $CDCl_3$): δ 12.45 (s, 1H), 8.02 (d, J = 8.3 Hz, 1H), 7.84 (d, J = 8.1 Hz, 1H), 7.80 (d, J = 5.9 Hz, 1H), 7.78 (s, 1H), 7.75 (d, J = 7.9 Hz, 2H), 7.58-7.64 (m, 4H), 7.48-7.52 (m, 1H), 7.44-7.48 (m, 2H), 7.37-7.42 (m, 1H), 7.25 (d, J = 8.4 Hz, 1H), 6.86 (d, J = 8.4 Hz, 1H), 6.34 (br. s., 1H), 5.04 (d, J = 5.1 Hz, 2H), 2.28 (s, 3H); ^{13}C NMR (150 MHz, $CDCl_3$): δ 192.3, 165.3, 160.6, 142.6, 136.8, 136.6, 134.9, 132.9, 132.1, 130.5, 128.3, 127.9, 127.9, 127.7 (2C), 127.0, 126.7 (2C), 126.1, 125.8, 125.1, 124.4, 122.4, 121.0, 118.5, 117.4, 41.5, 19.6; ESI-HRMS (-): *m/z* $[M-H]^-$ calcd for $C_{28}H_{22}NO_3^-$, 420.1605, found, 420.1620.

(E)-4-(3-(2-hydroxy-5-methylphenyl)-3-oxoprop-1-en-1-yl)-N-(2-methoxyphenyl)benzamide (1h)

The title compound **1h** was obtained by the reaction of compound **7a** with 2-methoxyaniline following the general procedure. Yellow solid, yield 51.6 %, mp 203-205 ℃. ^{1}H-NMR (600 MHz, DMSO-d_6): δ 12.30 (s, 1H), 9.60 (s, 1H), 8.16 (d, J = 15.6 Hz, 1H), 8.12 (d, J = 1.5 Hz, 1H), 8.05-8.08 (m, 4H), 7.89 (d, J = 15.4 Hz, 1H), 7.76 (dd, J = 1.1, 7.7 Hz, 1H), 7.41 (dd, J = 1.7, 8.3 Hz, 1H), 7.21 (td, J = 1.5, 8.1 Hz, 1H), 7.12 (dd, J = 1.0, 8.4 Hz, 1H), 6.99 (td, J = 1.1, 7.7 Hz, 1H), 6.93 (d, J = 8.4 Hz, 1H), 3.85 (s, 3H), 2.34 (s, 3H). ^{13}C-NMR (150 MHz, DMSO-d_6): 193.9, 164.8, 160.4, 152.2, 143.8, 137.9, 137.9, 136.5, 131.0, 129.6 (2C), 128.5, 128.5 (2C), 127.1, 126.5, 125.2, 124.0, 120.8, 120.7, 118.0, 111.9, 56.2, 20.4. ESI-HRMS (-): *m/z* $[M-H]^-$ calcd for $C_{24}H_{20}NO_4^-$, 386.1398, found, 386.1400.

(E)-N-(tert-butyl)-4-(3-(2-hydroxy-5-methylphenyl)-3-oxoprop-1-en-1-yl)benzamide (1i)

The title compound **1i** was obtained by the reaction of compound **7a** with *t*-butylamine following the general procedure. Yellow solid, yield 52.9 %, mp 177-179 ℃. ^{1}H NMR (600 MHz, $CDCl_3$): δ 12.48 (s, 1H), 7.83 (d, J = 15.6 Hz, 1H), 7.71 (d, J = 8.3 Hz, 2H), 7.61-7.65 (m, 4H), 7.26 (dd, J = 1.8, 8.4 Hz, 1H), 6.88 (d, J = 8.4 Hz, 1H), 5.91 (br. s., 1H), 2.29 (s, 3H), 1.42 (s, 9H). ^{13}C NMR (150 MHz, $CDCl_3$): δ 192.3, 165.0, 160.6, 142.8, 136.8, 136.6, 136.1, 128.3, 127.6 (2C), 127.0, 126.4 (2C), 120.7, 118.6, 117.4, 50.9, 27.8 (3C), 19.6. ESI-HRMS (-): *m/z* $[M-H]^-$ calcd for $C_{21}H_{22}NO_3^-$, 336.1605, found, 336.1615.

(E)-N-cyclobutyl-4-(3-(2-hydroxy-5-methylphenyl)-3-oxoprop-1-en-1-yl)benzamide (1j)

The title compound **1j** was obtained by the reaction of compound **7a** with cyclobutanamine following the general procedure. Yellow solid, yield 59.7 %, mp 209-211 ℃. ^{1}H NMR (600 MHz, $CDCl_3$): δ 12.55 (s, 1H), 7.89 (d, J = 15.6 Hz, 1H), 7.82 (d, J = 8.3 Hz, 2H), 7.67-7.73 (m, 4H), 7.33 (dd, J = 1.8, 8.4 Hz, 1H), 6.94 (d, J = 8.4 Hz, 1H), 6.40 (br. s., 1H), 4.61 (sxt, J = 8.1 Hz, 1H), 2.42-2.49 (m, 2H), 2.36 (s, 3H), 1.95-2.04 (m, 2H), 1.75-1.84 (m, 2H). ^{13}C NMR (150 MHz, $CDCl_3$): δ 193.3, 165.7, 161.6, 143.7, 137.8, 137.4, 136.3, 129.3, 128.7 (2C), 128.1 , 127.6 (2C), 121.9, 119.6, 118.5, 45.3, 31.3 (2C), 20.6, 15.2. ESI-HRMS (-): *m/z* $[M-H]^-$ calcd for $C_{21}H_{20}NO_3^-$, 334.1449, found, 334.1435.

(E)-N-hexyl-4-(3-(2-hydroxy-5-methylphenyl)-3-oxoprop-1-en-1-yl)benzamide (1k)

The title compound **1k** was obtained by the reaction of compound **7a** with hexanamine following the general procedure. Yellow solid, yield 42.9 %, mp 152-153 ℃. ^{1}H NMR (600 MHz, $CDCl_3$): δ 12.48 (s, 1H), 7.82 (d, J = 15.6 Hz, 1H), 7.75 (d, J = 8.3 Hz, 2H), 7.59-7.65 (m, 4H), 7.26 (dd, J = 1.8, 8.4 Hz, 1H), 6.87 (d, J = 8.6 Hz, 1H), 6.19 (br. s., 1H), 3.37-3.42 (m, 2H), 2.29 (s, 3H), 1.56 (quin, J = 7.43 Hz, 2H), 1.29-1.35 (m, 2H), 1.23-1.28 (m, 4H), 0.80-0.85 (m, 3H). ^{13}C NMR (150 MHz, $CDCl_3$): δ 192.3, 165.6, 160.6, 142.7, 136.8, 136.4, 135.5, 128.3, 127.7 (2C), 127.1, 126.5 (2C), 120.8, 118.5, 117.4, 39.2, 30.5, 28.6, 25.7, 21.5, 19.6, 13.0. ESI-HRMS (-): *m/z* $[M-H]^-$ calcd for $C_{23}H_{26}NO_3^-$, 364.1918, found, 364.1915.

(E)-4-(3-(2-hydroxy-5-methylphenyl)-3-oxoprop-1-en-1-yl)-N-octylbenzamide (1l)

The title compound **1l** was obtained by the reaction of compound **7a** with octylamine following the general procedure. Yellow solid, yield 54.7 %, mp 121-123 ℃. ^{1}H NMR (600 MHz, $CDCl_3$): δ 12.48 (s, 1H), 7.83 (d, J = 15.4 Hz, 1H), 7.75 (d, J = 8.1 Hz, 2H), 7.61-7.67 (m, 4H), 7.25 (dd, J = 1.8, 8.4 Hz, 1H), 6.88 (d, J = 8.4 Hz, 1H), 6.10 (br. s., 1H), 3.39-3.42 (m, 2H), 2.30 (s, 3H), 1.51-1.60 (m, 2H), 1.20-1.35 (m, 10H), 0.82 (t, J = 6.7 Hz, 3H). ^{13}C NMR (150 MHz, $CDCl_3$): δ 192.3, 165.7, 160.6, 142.7, 136.8, 136.4, 135.5, 128.3, 127.7 (2C), 127.0, 126.5 (2C), 120.9, 118.5, 117.4, 39.3, 30.8, 28.6, 28.3, 28.2, 26.0, 21.6, 19.6, 13.1. ESI-HRMS (-): *m/z* $[M-H]^-$ calcd for $C_{25}H_{30}NO_3^-$, 392.2231, found, 392.2205.

(E)-N-cyclooctyl-4-(3-(2-hydroxy-5-methylphenyl)-3-oxoprop-1-en-1-yl)benzamide (1m)

The title compound **1m** was obtained by the reaction of compound **7a** with cyclooctanamine

following the general procedure. Yellow solid, yield 55.8 %, mp 200-202 ℃. ^{1}H NMR (600 MHz, $CDCl_3$): δ 12.48 (s, 1H), 7.82 (d, *J* = 15.6 Hz, 1H), 7.73 (d, *J* = 8.3 Hz, 2H), 7.60-7.66 (m, 4H), 7.26 (dd, *J* = 1.8, 8.4 Hz, 1H), 6.87 (d, *J* = 8.4 Hz, 1H), 6.06 (br. s., 1H), 4.10-4.18 (m, 1H), 2.29 (s, 3H), 1.85-1.91 (m, 2H), 1.46-1.64 (m, 12H). ^{13}C NMR (150 MHz, $CDCl_3$): δ 192.3, 164.4, 160.6, 142.7, 136.8, 136.3, 135.8, 128.3, 127.6 (2C), 127.0, 126.5 (2C), 120.8, 118.5, 117.4, 49.0, 31.4 (2C), 26.1 (2C), 24.5, 22.7 (2C), 19.6; ESI-HRMS (-): *m/z* $[M-H]^-$ calcd for $C_{25}H_{28}NO_3^-$, 390.2075, found, 390.2115.

N-(adamantan-1-yl)-4-((E)-3-(2-hydroxy-5-methylphenyl)-3-oxoprop-1-en-1-yl)benzamide (1n)

The title compound **1n** was obtained by the reaction of compound **7a** with amantadine following the general procedure. Yellow solid, yield 48.3 %, mp 236-238 ℃. ^{1}H NMR (600 MHz, $CDCl_3$): δ 12.49 (s, 1H), 7.83 (d, *J* = 15.4 Hz, 1H), 7.71 (d, *J* = 7.5 Hz, 2H), 7.61-7.66 (m, 4H), 7.27 (dd, *J* = 2.0, 8.4 Hz, 1H), 6.88 (d, *J* = 8.4 Hz, 1H), 5.75 (br. s., 1H), 2.30 (s, 3H), 2.09-2.06 (m, 9H), 1.68-1.65 (m, 6H). ^{13}C NMR (150 MHz, $CDCl_3$): δ 192.3, 164.7, 160.6, 142.8, 136.7, 136.6, 136.1, 128.3, 127.6 (2C), 127.0, 126.4 (2C), 120.7, 118.6, 117.4, 51.5, 40.6 (3C), 35.3 (3C), 28.5 (3C), 19.6. ESI-HRMS (-): *m/z* $[M-H]^-$ calcd for $C_{27}H_{28}NO_3^-$, 414.2075, found, 414.2103.

(E)-3-(3-(2-hydroxy-5-methylphenyl)-3-oxoprop-1-en-1-yl)-N-(2-(thiophen-3-yl)ethyl)benzamide (1o)

The title compound **1o** was obtained by the reaction of compound **7b** with 3-thiopheneethanamine following the general procedure. Yellow solid, yield 44.6 %, mp 135-137 ℃. ^{1}H-NMR (600 MHz, $CDCl_3$): δ 12.50 (s, 1H), 8.04 (s, 1H), 7.80 (d, *J* = 15.6 Hz, 1H), 7.66 (d, *J* = 7.7 Hz, 1H), 7.61-7.65 (m, 3H), 7.40 (t, *J* = 7.7 Hz, 1H), 7.25 (dd, *J* = 1.8, 8.4 Hz, 1H), 7.12 (dd, *J* = 0.7, 5.1 Hz, 1H), 6.91 (dd, *J* = 3.4, 5.0 Hz, 1H), 6.86 (d, *J* = 8.4 Hz, 1H), 6.82 (d, *J* = 3.3 Hz, 1H), 6.37 (br. s., 1H), 3.69 (q, *J* = 6.4 Hz, 2H), 3.12 (t, *J* = 6.6 Hz, 2H), 2.29 (s, 3H); ^{13}C NMR (150 MHz, $CDCl_3$): δ 193.4, 167.0, 161.5, 143.9, 141.1, 137.8, 135.4, 135.1, 131.9, 129.4, 129.3, 128.7, 128.1, 127.2, 126.8, 125.5, 124.1, 121.3, 119.6, 118.3, 41.5, 29.8, 20.6; ESI-HRMS (+): *m/z* $[M+Na]^+$ calcd for $C_{23}H_{21}NO_3SNa^+$, 414.1134, found, 414.1129.

(E)-3-(3-(2-hydroxy-5-methylphenyl)-3-oxoprop-1-en-1-yl)-N-phenethylbenzamide (1p)

The title compound **1p** was obtained by the reaction of compound **7b** with 2-phenylethanamine following the general procedure. Yellow solid, yield 41.3 %, mp 151-153 ℃. ^{1}H-NMR (600 MHz, $CDCl_3$): δ 12.50 (s, 1H), 8.01 (s, 1H), 7.79 (d, J = 15.4 Hz, 1H), 7.65 (d, J = 7.7 Hz, 1H), 7.60-7.63 (m, 2H), 7.58 (d, J = 7.9 Hz, 1H), 7.39 (t, J = 7.6 Hz, 1H), 7.24-7.28 (m, 3H), 7.16-7.20 (m, 3H), 6.86 (d, J = 8.6 Hz, 1H), 6.21 (br. s., 1H), 3.68 (q, J = 6.8 Hz, 2H), 2.89 (t, J = 6.9 Hz, 2H), 2.29 (s, 3H); ^{13}C-NMR (150 MHz, $CDCl_3$): δ 192.3, 165.9, 160.5, 142.9, 137.8, 136.7, 134.4, 134.0, 130.8, 128.4, 128.2, 127.8 (2C), 127.7 (2C), 127.6, 127.1, 125.7, 125.6, 120.2, 118.5, 117.3, 40.3, 34.6, 19.5; ESI-HRMS (+): *m/z* $[M+Na]^+$ calcd for $C_{25}H_{23}NO_3Na^+$, 408.1570, found 408.1565.

3-(Adamant-1-yl)-4-hydroxybenzaldehyde (9a)

To a solution of 4-hydroxybenzaldehyde **8a** (1.22 g, 10 mmol) in CH_2Cl_2 (40 mL) was added adamant-1-ol (1.69 g, 11.1 mmol) and H_2SO_4 (0.65 mL, 12 mmol). The reaction mixture was stirred at 50 ℃ for 16 h. After addition of a saturated aqueous $NaHCO_3$ solution until neutral pH was reached, the mixture was extracted with CH_2Cl_2 (3 × 100 mL). The combined organic layer was washed with water, brine solution, dried over anhydrous Na_2SO_4 and the organic solvent was evaporated. The residue was purified by column chromatography using hexane/EtOAc (20:1) to afford **9a**. White solid, yield 53.1 %. ^{1}H NMR (600 MHz, DMSO-d_6): δ 9.78 (s, 1H), 7.66 (d, J = 2.1 Hz, 1H), 7.60 (dd, J = 2.1, 8.3 Hz, 1H), 6.94 (d, J = 8.3 Hz, 1H), 2.04-2.08 (m, 9H), 1.72-1.76 (m, 6H). ^{13}C NMR (150 MHz, DMSO-d_6): δ 191.6, 160.6, 137.2, 129.9, 129.7, 128.2, 117.3, 40.3 (3C), 37.0 (3C), 36.9, 28.9 (3C).

3-(Adamant-1-yl)-4-(methoxymethoxy)-benzaldehyde (10a)

To a suspension of NaH (0.41 g, 60% in mineral oil, 10.2 mmol) in DMF (7 mL) was added a solution of 3-(adamant-1-yl)-4-hydroxybenzaldehyde **9a** (1.04 g, 4.06 mmol) in DMF (4 mL) at 0 ℃ under a nitrogen atmosphere and the reaction was stirred for 30 min. Then chloromethyl methyl ether (MOMCl) (1.0 mL, 8.12 mmol) was added and the reaction mixture was stirred at room temperature for 19 h. After completion of the reaction, the resulting mixture was poured into iced-water and extracted with ethyl acetate (3 × 50 mL).

The combined organic layer was washed with water, brine solution, dried over anhydrous Na_2SO_4 and the organic solvent was evaporated under reduced pressure. The residue was purified by column chromatography using hexane/EtOAc (20:1) to afford **10a**. White solid, yield 88.1 %. ^{1}H NMR (600 MHz, $CDCl_3$): δ 9.91 (s, 1H), 7.83 (d, J = 2.0 Hz, 1H), 7.72 (dd, J = 2.0, 8.3 Hz, 1H), 7.22 (d, J = 8.3 Hz, 1H), 5.33 (s, 2H), 3.55 (s, 3H), 2.11-2.16 (m, 9H), 1.80-1.82 (m, 6H). ^{13}C NMR (150 MHz, $CDCl_3$): δ 191.5, 161.4, 139.3, 130.3, 130.0, 128.6, 114.3, 94.0, 56.6, 40.5 (3C), 37.3 (3C), 37.0, 28.9 (3C).

5-(Adamant-1-yl)-2,4-dihydroxybenzaldehyde (9b)[3]

Following the same procedure for **9a**, **9b** was obtained from 2,4-dihydroxybenzaldehyde and adamant-1-ol**.** White solid, yield 76.8 %. ^{1}H NMR (600 MHz, $CDCl_3$): δ 11.24 (s, 1H), 9.73 (s, 1H), 7.36 (s, 1H), 6.26 (s, 1H), 2.10-2.13 (m, 9H), 1.79-1.82 (m, 6H). ^{13}C NMR (150 MHz, $CDCl_3$): δ 193.8, 161.5, 161.0, 132.1, 128.9, 114.1, 103.3, 39.7 (3C), 35.9 (3C), 35.2, 27.9 (3C).

5-(Adamant-1-yl)-2,4-bis(methoxymethoxy)-benzaldehyde (10b)

Following the same procedure for **10a**, **10b** was obtained from **9b** and MOMCl. White solid, yield 90.1 %. ^{1}H NMR (600 MHz, $CDCl_3$): δ 10.34 (s, 1H), 7.74 (s, 1H), 6.90 (s, 1H), 5.29 (d, J = 8.8 Hz, 4H), 3.53 (d, J = 5.0 Hz, 6H), 2.07-2.10 (m, 9H), 1.75-1.78 (m, 6H). ^{13}C NMR (150 MHz, $CDCl_3$): δ 188.5, 162.5, 159.7, 132.9, 126.8, 119.1, 101.0, 94.8, 94.1, 56.7, 56.5, 40.7 (3C), 37.0 (3C), 36.7, 29.0 (3C).

(E)-3-(3-(adamantan-1-yl)-4-(methoxymethoxy)phenyl)-1-(2-hydroxyphenyl)prop-2-en-1-one (3a)

Following the general procedure for the Claisen-Schmidt condensation, 3-(adamant-1-yl)-4-(methoxymethoxy)-benzaldehyde **10a** (1 mmol, 300.2 mg) and 1-(2-hydroxyphenyl)ethanone (1 mmol, 136.0 mg) were used to give **3a** (224.5 mg, 53.7 %) as a yellow solid. Mp: 158-162 ℃. ^{1}H NMR (600 MHz, $CDCl_3$): δ 12.98 (s, 1H), 7.94 (dd, J = 1.5, 8.0 Hz, 1H), 7.92 (d, J = 15.6 Hz, 1H), 7.50-7.55 (m, 2H), 7.44-7.50 (m, 2H), 7.12 (d, J = 8.5 Hz, 1H), 7.01 (dd, J = 0.9, 8.3 Hz, 1H), 6.91-6.96 (m, 1H), 5.27 (s, 2H), 3.52 (s, 3H), 2.08-2.17 (m, 9H), 1.78-1.82 (m, 6H); ^{13}C NMR (150 MHz, $CDCl_3$): δ 193.7, 163.6, 158.9, 146.2, 139.2, 136.1, 129.6, 128.0, 127.9, 127.9, 120.2, 118.7, 118.6, 117.5, 114.7, 94.1, 56.5, 40.6 (3C), 37.2, 37.1 (3C), 29.0 (3C); ESI-HRMS (-): m/z $[M-H]^-$ calcd for $C_{27}H_{29}O_4^-$,

417.2071, found, 417.2073.

(E)-3-(3-(adamantan-1-yl)-4-(methoxymethoxy)phenyl)-1-(5-chloro-2-hydroxyphenyl)prop-2-en-1-one (3b)

Following the general procedure for the Claisen-Schmidt condensation, 3-(adamant-1-yl)-4-(methoxymethoxy)-benzaldehyde **10a** (1 mmol, 300.2 mg) and 1-(5-chloro-2-hydroxyphenyl)ethanone (1 mmol, 170.0 mg) were used to give **3b** (226.6 mg, 50.1 %) as a yellow solid. Mp: 122-123 ℃. ^{1}H NMR (600 MHz, $CDCl_3$): δ 12.89 (s, 1H), 7.93 (d, J = 15.3 Hz, 1H), 7.87 (d, J = 1.8 Hz, 1H), 7.49-7.56 (m, 2H), 7.38-7.45 (m, 2H), 7.15 (d, J = 8.3 Hz, 1H), 6.97 (d, J = 9.0 Hz, 1H), 5.29 (s, 2H), 3.54 (s, 3H), 2.09-2.18 (m, 9H), 1.78-1.83 (m, 6H); ^{13}C NMR (150 MHz, $CDCl_3$): δ 191.8, 161.0, 158.1, 146.3, 138.3, 134.8, 127.7, 127.4, 127.0, 126.6, 122.3, 119.8, 119.1, 115.8, 113.8, 93.0, 55.5, 39.5 (3C), 36.2, 36.0 (3C), 28.0 (3C); ESI-HRMS (-): m/z $[M-H]^-$ calcd for $C_{27}H_{28}ClO_4^-$, 451.1682, found, 451.1679.

(E)-3-(3-(adamantan-1-yl)-4-(methoxymethoxy)phenyl)-1-(2-hydroxy-5-methylphenyl)prop-2-en-1-one (3c)

Following the general procedure for the Claisen-Schmidt condensation, 3-(adamant-1-yl)-4-(methoxymethoxy)-benzaldehyde **10a** (1 mmol, 300.2 mg) and 1-(2-Hydroxy-5-methylphenyl)ethanone (1 mmol, 150.1 mg) were used to give **3c** (199.8 mg, 51.5 %) as a yellow solid. Mp: 123-125 ℃. ^{1}H NMR (600 MHz, $CDCl_3$): δ 12.79 (s, 1H), 7.89 (d, J = 15.3 Hz, 1H), 7.68 (d, J = 1.3 Hz, 1H), 7.52-7.53 (m, 2H), 7.48-7.51 (m, 1H), 7.28 (dd, J = 2.0, 8.5 Hz, 1H), 7.13 (d, J = 8.3 Hz, 1H), 6.92 (d, J = 8.3 Hz, 1H), 5.27 (s, 2H), 3.52 (s, 3H), 2.35 (s, 3H), 2.11-2.15 (m, 9H), 1.78-1.82 (m, 6H). ^{13}C NMR (150 MHz, $CDCl_3$): δ 193.7, 161.5, 158.8, 146.1, 139.2, 137.2, 129.3, 128.3, 128.0, 127.8, 127.6, 119.8, 118.3, 117.7, 114.8, 94.1, 56.5, 40.6 (3C), 37.2, 37.1 (3C), 29.0 (3C), 20.7. ESI-HRMS (-): m/z $[M-H]^-$ calcd for $C_{28}H_{31}O_4^-$, 431.2301, found, 431.2306.

(E)-3-(5-(adamantan-1-yl)-2,4-bis(methoxymethoxy)phenyl)-1-(2-hydroxy-5-methylphenyl)prop-2-en-1-one (3d, Amt-87)

Following the general procedure for the Claisen-Schmidt condensation, 5-(adamantan-1-yl)-2,4-bis(methoxymethoxy)benzaldehyde **10b** (1 mmol, 360.2 mg) and 1-(2-hydroxy-5-methylphenyl)ethanone (1 mmol, 150.1 mg) were used to give Amt-87

(261.7 mg, 53.2 %) as a yellow solid. Mp: 114-116 ℃. ^{1}H NMR (600 MHz, DMSO-d_6): δ 12.44 (s, 1H), 8.15 (d, J = 15.6 Hz, 1H), 7.94 (s, 1H), 7.83 (d, J = 15.6 Hz, 1H), 7.61 (s, 1H), 7.36 (d, J = 8.3 Hz, 1H), 6.89 (d, J = 8.3 Hz, 1H), 6.86 (s, 1H), 5.33 (s, 2H), 5.32 (s, 2H), 3.47 (s, 3H), 3.45 (s, 3H), 2.32 (s, 3H), 2.06-2.13 (m, 9H), 1.74-1.77 (m, 6H). ^{13}C NMR (150 MHz, $CDCl_3$): δ 194.0, 160.2, 159.7, 156.3, 140.4, 137.2, 132.5, 130.5, 128.2, 127.2, 121.1, 119.5, 118.0, 116.8, 102.1, 95.2, 94.5, 56.9, 56.6, 40.7 (3C), 37.0 (3C), 36.7, 28.9 (3C), 20.5. ESI-HRMS (-): *m/z* [M-H]$^-$ calcd for $C_{30}H_{35}O_6^-$, 491.2439, found, 491.2430.

(E)-3-(5-(adamantan-1-yl)-2,4-bis(methoxymethoxy)phenyl)-1-(m-tolyl)prop-2-en-1-one (3e)

Following the general procedure for the Claisen-Schmidt condensation, 5-(adamantan-1-yl)-2,4-bis(methoxymethoxy)benzaldehyde **10b** (1 mmol, 360.2 mg) and 3'-methylacetophenonene (1 mmol, 134.1 mg) were used to give **3e** (267.7 mg, 56.2 %) as a yellow solid. Mp: 110-112 ℃. ^{1}H NMR (600 MHz, $CDCl_3$): δ 8.02 (d, J = 15.8 Hz, 1H), 7.74 (s, 1H), 7.72 (d, J = 5.7 Hz, 1H), 7.39-7.45 (m, 2H), 7.27-7.31 (m, 2H), 6.85 (s, 1H), 5.17 (s, 2H), 5.16 (s, 2H), 3.45 (s, 3H), 3.43 (s, 3H), 2.36 (s, 3H), 1.96-2.05 (m, 9H), 1.68-1.73 (m, 6H); ^{13}C NMR (150 MHz, $CDCl_3$): δ 191.5, 159.4, 156.0, 140.7, 138.9, 138.3, 133.1, 132.7, 129.0, 128.3, 127.2, 125.7, 120.7, 117.4, 101.9, 94.9, 94.3, 56.6, 56.5, 40.9 (3C), 37.1 (3C), 36.7, 29.1 (3C), 21.4; ESI-HRMS (+): *m/z* [M+Na]$^-$ calcd for $C_{30}H_{36}O_5Na^+$, 499.2455, found, 499.2453.

General procedure of the synthesis of 4a-4d.

To a stirred solution of **3 (3a-3d)** (1 equiv) in MeOH (5 mL) was added dropwise of HCl (10% aqueous solution, 2.0 mL). The mixture was refluxed at 70 ℃ for 1 h. After cooling to room temperature, the mixture was diluted with water (10 mL) and extracted with ethyl acetate (3×10 mL). The combined organic layer was dried with anhydrous Mg_2SO_4, filtered, and concentrated. The residue was purified by column chromatography over silica gel to obtain the corresponding compound.

(E)-3-(3-(adamantan-1-yl)-4-hydroxyphenyl)-1-(2-hydroxyphenyl)prop-2-en-1-one (4a)

Yield: 30.3 %, mp: 180-183 ℃. ^{1}H NMR (600 MHz, DMSO-d_6): δ 12.84 (s, 1H), 10.16 (s, 1H), 8.26 (dd, J = 1.5, 8.0 Hz, 1H), 7.83 (d, J = 15.6 Hz, 1H), 7.78 (d, 1H, J = 15.6 Hz, 1H), 7.63 (dd, J = 2.0, 8.3 Hz, 1H), 7.52-7.58 (m, 2H), 6.97-7.03 (m, 2H), 6.87 (d, J = 8.3 Hz,

1H), 2.06-2.12 (m, 9H), 1.77-1.80 (m, 6H); ^{13}C NMR (150 MHz, DMSO-d$_6$): δ 194.0, 162.5, 160.1, 147.0, 136.7, 136.5, 131.1, 129.1, 129.0, 125.8, 121.1, 119.5, 118.2, 117.6, 117.5, 39.0 (3C), 37.1 (3C), 36.5, 28.8 (3C); ESI-HRMS (-): *m/z* [M-H]$^-$ calcd for $C_{25}H_{25}O_3^-$, 373.1804, found, 373.1808.

(E)-3-(3-(adamantan-1-yl)-4-hydroxyphenyl)-1-(5-chloro-2-hydroxyphenyl)prop-2-en-1-one (4b)

Yield 34.1 %, mp 185-187 ℃. ^{1}H NMR (600 MHz, DMSO-d$_6$): δ 12.65 (s, 1H), 10.17 (s, 1H), 8.24 (d, *J* = 2.5 Hz, 1H), 7.82 (d, *J* = 15.6 Hz, 1H), 7.75 (d, *J* = 15.6 Hz, 1H), 7.69 (dd, *J* = 1.8, 8.5 Hz, 1H), 7.53 (dd, *J* = 2.5, 8.8 Hz, 1H), 7.50 (d, *J* = 1.8 Hz, 1H), 7.01 (d, *J* = 8.8 Hz, 1H), 6.88 (d, *J* = 8.3 Hz, 1H), 2.04-2.10 (m, 9H), 1.70-1.73 (m, 6H); ^{13}C NMR (150 MHz, DMSO-d$_6$): δ 192.8, 160.6, 160.2, 147.7, 136.7, 135.6, 129.9, 129.6, 128.9, 125.8, 123.2, 122.8, 120.1, 118.0, 117.5, 40.2 (3C), 37.1 (3C), 36.8, 28.8 (3C); ESI-HRMS (-): *m/z* [M-H]$^-$ calcd for $C_{25}H_{24}ClO_3^-$, 407.1414, found, 407.1417.

(E)-3-(3-(adamantan-1-yl)-4-hydroxyphenyl)-1-(2-hydroxy-5-methylphenyl)prop-2-en-1-one (4c)

Yield 37.4 %, mp 174-175 ℃. ^{1}H NMR (600 MHz, DMSO-d$_6$): δ 12.64 (s, 1H), 10.14 (s, 1H), 8.04 (d, *J* = 1.3 Hz, 1H), 7.82 (d, *J* = 15.3 Hz, 1H), 7.78 (d, *J* = 15.6 Hz, 1H), 7.70 (dd, *J* = 1.9, 8.4 Hz, 1H), 7.50 (d, *J* = 2.0 Hz, 1H), 7.36 (dd, *J* = 1.8, 8.3 Hz, 1H), 6.88 (dd, *J* = 1.4, 8.4 Hz, 2H), 2.33 (s, 3H), 2.02-2.14 (m, 9H), 1.73-1.76 (m, 6H). ^{13}C NMR (150 MHz, DMSO-d$_6$): δ 193.9, 160.4, 160.0, 146.8, 137.3, 136.7, 130.6, 129.5, 128.5, 128.3, 125.9, 120.7, 118.0, 117.8, 117.5, 40.6 (3C), 37.1 (3C), 36.8, 28.8 (3C), 20.5. ESI-HRMS (-): *m/z* [M-H]$^-$ calcd for $C_{26}H_{27}O_3^-$, 387.1966, found, 387.1964.

(E)-3-(5-(adamantan-1-yl)-2,4-dihydroxyphenyl)-1-(2-hydroxy-5-methylphenyl)prop-2-en-1-one (4d)

Yield 37.4 %, mp 154-157 ℃. ^{1}H NMR (600 MHz, DMSO-d$_6$): δ 12.83 (s, 1H), 10.1 (br. s., 2H), 8.14 (d, *J* = 15.3 Hz, 1H), 7.88 (s, 1H), 7.73 (d, *J* = 15.3 Hz, 1H), 7.40 (s, 1H), 7.31 (dd, *J* = 1.1, 8.4 Hz, 1H), 6.86 (d, *J* = 8.3 Hz, 1H), 6.47 (s, 1H), 2.29 (s, 3H), 1.98-2.08 (m, 9H), 1.68-1.73 (m, 6H); ^{13}C NMR (150 MHz, DMSO-d$_6$): δ 194.0, 161.5, 160.5, 158.1, 142.8, 136.9, 130.1, 129.2, 128.2, 128.0, 120.8, 118.0, 116.0, 113.0, 104.2, 40.7(3C), 37.1(3C), 36.2, 28.9(3C), 20.5; ESI-HRMS (-): *m/z* [M-H]$^-$ calcd for $C_{26}H_{27}O_4^-$, 404.1915, found,

404.1915.

References

1. Chen Y.P., et al., *Syntheses and evaluation of novel isoliquiritigenin derivatives as potential dual inhibitors for amyloid-Beta aggregation and 5-lipoxygenase*. European Journal of Medicinal Chemistry, 2013. **44**(52): p. 22-31.
2. Tu H.Y., et al. *Synthesis and biological evaluation of 2',5'-dimethoxychalcone derivatives as microtubule-targeted anticancer agents*. Bioorganic & Medicinal Chemistry, 2010. **18**(6): p. 2089-2098.
3. Lorenzo P., et al., *Inhibition of IκB kinase-beta and anticancer activities of novel chalcone adamantyl arotinoids.* Journal of Medicinal Chemistry, 2008. **51**(17): p. 5431-5440.
4. Zhang B., et al., *Synthesis of xanthohumol analogues and discovery of potent thioredoxin reductase inhibitor as potential anticancer agent.* Journal of Medicinal Chemistry, 2015. **58**(4): p. 1795-1805.

Supplemetary Figure

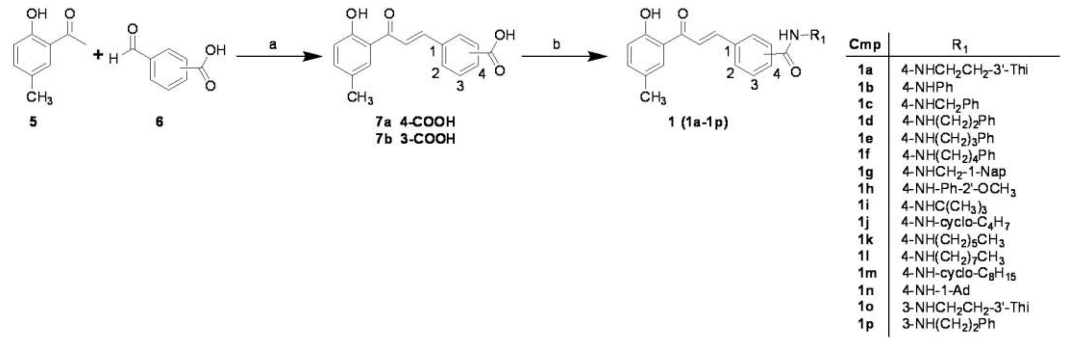

Figure S1. Synthesis of 2'-hydroxy-chalcone amide derivatives 1a-1p

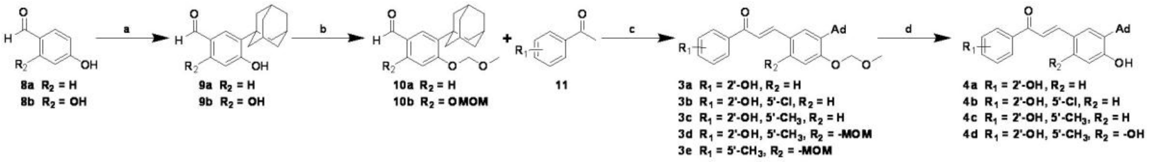

Figure S2. Synthesis of 5-adamantyl-chalcones 3a-3e and 4a-4d.

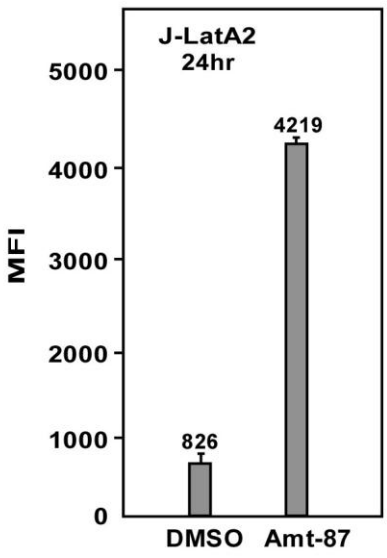

Figure S3. The mean fluorescence intensity (MFI) values of GFP-positive cells in reactivated J-Lat A2 population.

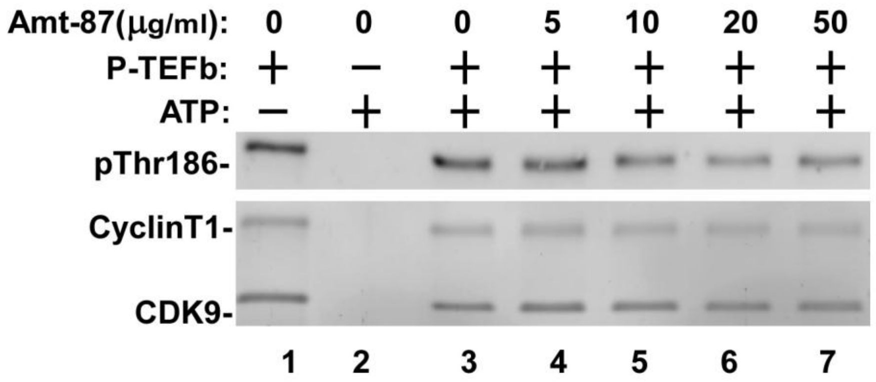

Figure S4. Amt-87 does not affect the phosphorylation of CDK9 T-loop in vitro.

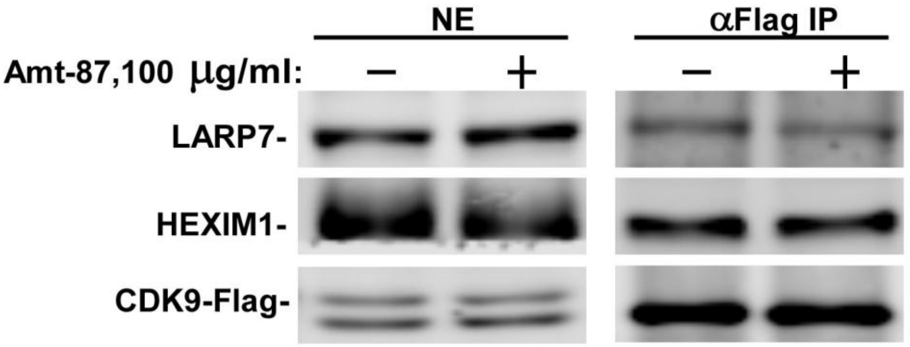

Figure S5. Amt-87 does not cause 7SK snRNP dissociation in vitro.

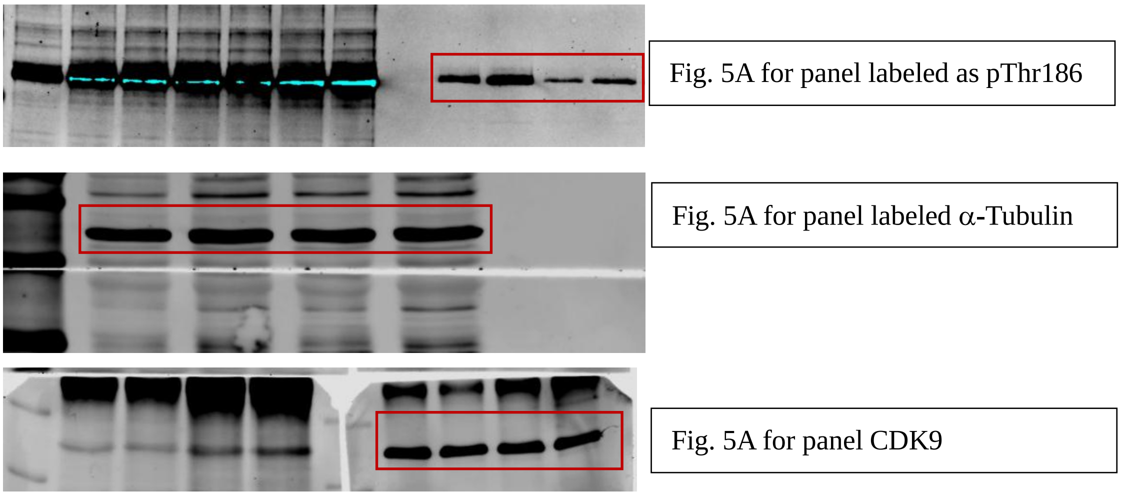

Fig. S6. Scans of original films for making Figure 5A

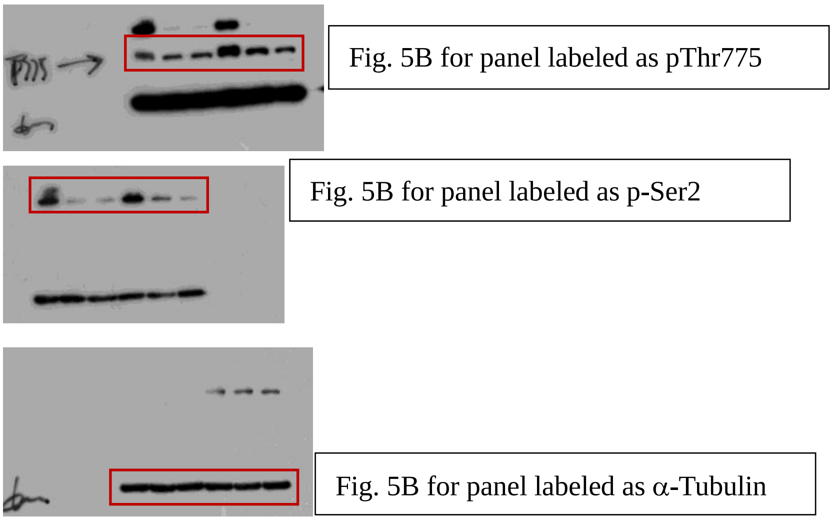

Fig. S7. Scans of original films for making Figure 5B

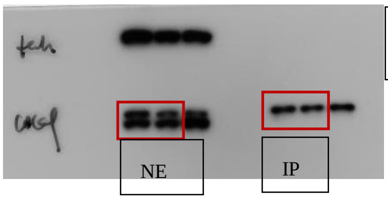

Fig. 6A for panel labeled as CDK9

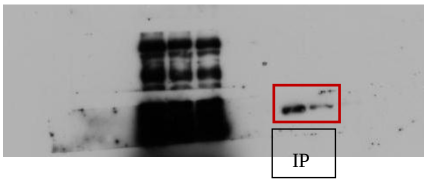

Fig. 6A for panel HEXIM1, IP

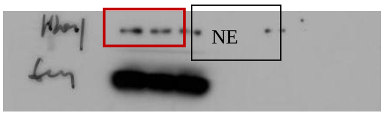

Fig. 6A for panel HEXIM1, NE

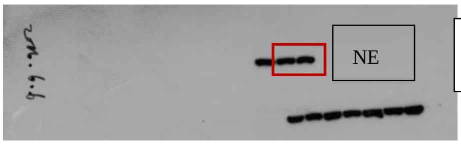

Fig. 6A for a-Tubulin, NE

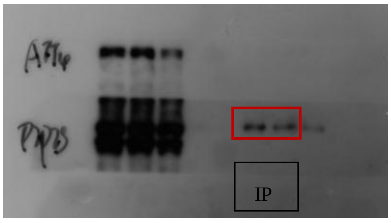

Fig. 6A for panel LARP7, IP

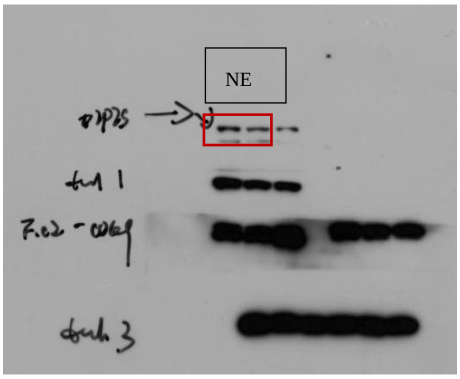

Fig. 6A for panel LARP7, NE

Fig. S8. Scans of original films for making Figure 6A

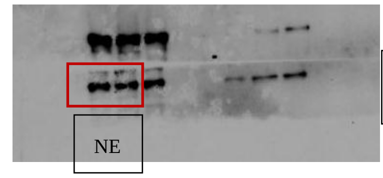

Fig. 6B for panel labeled as ELL2, NE

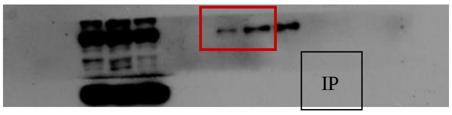

Fig. 6B for panel ELL2, IP

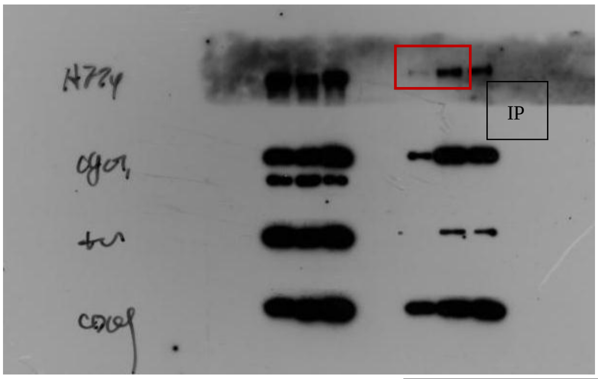

Fig. 6B for panel AFF4, IP

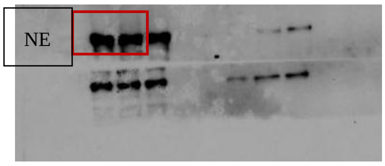

Fig. 6B for panel AFF4, NE

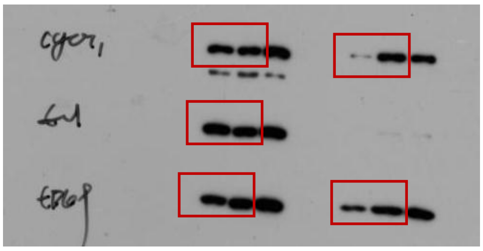

Fig. 6B for panel CyclinT1

Fig. 6B for panel α-Tubulin

Fig. 6B for panel CDK9

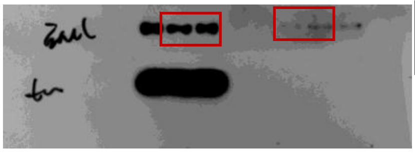

Fig. 6B for panel ENL

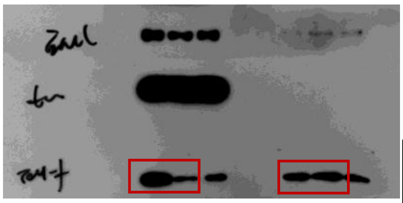

Fig. 6B for panel Tat-Flag

Fig. S9. Scans of original films for making Figure 6B

Supplementary Figure legends

Figure S1. Synthesis of 2'-hydroxy-chalcone amide derivatives 1a-1p. Reagents and conditions: (a) NaOH, EtOH, r.t., 24 hr. (b) various amines, anhydrous HOBt, EDCI, DCM, 12 hr.

Figure S2. Synthesis of 5-adamantyl-chalcones 3a-3e and 4a-4d. Reagents and conditions: (a) adamant-1-ol, H_2SO_4, CH_2Cl_2, 50 ℃, 16 hr; (b) NaH, MOMCl, DMF, 19 hr; (c) NaOH, EtOH, r.t., 24 hr. (d) HCl, MeOH, reflux, 1 hr.

Figure S3. The mean fluorescence intensity (MFI) values of GFP-positive cells in reactivated J-Lat A2 population. J-Lat A2 cells were treated with DMSO or 200 μg/ml Amt-87 for 24 hr. The MFI values were determined by evaluating the FACS plots.

Figure S4. Amt-87 does not affect the phosphorylation of CDK9 T-loop in vitro. Kinase reactions (25 μl) containing 40 μM ATP, 30 ng affinity-purified P-TEFb, 30 ng GST-CTD and the indicated concentrations of Amt-87 were performed in kinase reaction buffer (50 mM Hepes, pH7.4, 10 mM $MgCl_2$, 50 mM NaCl, and 1 mM DTT) for 30 min at 30 ℃. The reaction products were analyzed by Western blotting to detect the levels of CDK9, CycT1 and pThr186 on CDK9.

Figure S5. Amt-87 does not cause 7SK snRNP dissociation in vitro. Nuclear extracts (NE) of F1C2 cells that stably express CDK9-Flag and anti-Flag immunoprecipitates that were derived from NE and still attached to the Flag beads were incubated with DMSO (-) or 100 μg/ml Amt-87 at room temperature for 30 min. After washing, CDK9-Flag and its associated proteins were eluted off the beads and analyzed by Western blotting for the indicated 7SK snRNP components.
